# Association of Outdoor Ambient Fine Particulate Matter With Intracellular White Matter Microstructural Properties Among Children

**DOI:** 10.1001/jamanetworkopen.2021.38300

**Published:** 2021-12-09

**Authors:** Elisabeth Burnor, Dora Cserbik, Devyn L. Cotter, Clare E. Palmer, Hedyeh Ahmadi, Sandrah P. Eckel, Kiros Berhane, Rob McConnell, Jiu-Chiuan Chen, Joel Schwartz, Raymond Jackson, Megan M. Herting

**Affiliations:** 1 Department of Population and Public Health Sciences, Keck School of Medicine of University of Southern California, Los Angeles; 2 Center for Human Development, University of California, San Diego, La Jolla; 3 Department of Biostatistics, Columbia University Mailman School of Public Health, New York, New York; 4 Department of Neurology, Keck School of Medicine of University of Southern California, Los Angeles; 5 Department of Environmental Health, Harvard T.H. Chan School of Public Health, Boston, Massachusetts; 6 Children’s Hospital Los Angeles, Los Angeles, California

## Abstract

**Importance:**

Outdoor particulate matter 2.5 μm or less in diameter (PM_2.5_) is a ubiquitous environmental neurotoxicant that may affect the developing brain. Little is known about associations between PM_2.5_ and white matter connectivity.

**Objectives:**

To assess associations between annual residential PM_2.5_ exposure and white matter microstructure health in a US sample of children 9 to 10 years of age and to examine whether associations are specific to certain white matter pathways or vary across neuroimaging diffusion markers reflective of intracellular and extracellular microstructural processes.

**Design, Setting, and Participants:**

This cross-sectional study, the Adolescent Brain and Cognitive Development (ABCD) Study, was composed of 21 study sites across the US and used baseline data collected from children 9 to 10 years of age from September 1, 2016, to October 15, 2018. Data analysis was performed from September 15, 2020, to June 30, 2021.

**Exposures:**

Annual mean PM_2.5_ exposure estimated by ensemble-based models and assigned to the primary residential addresses at baseline.

**Main Outcomes and Measures:**

Diffusion-weighted imaging (DWI) and tractography were used to delineate white matter tracts. The biophysical modeling technique of restriction spectrum imaging (RSI) was implemented to examine total hindered diffusion and restricted isotropic and anisotropic intracellular diffusion in each tract. Hierarchical mixed-effects models with natural splines were used to analyze the associations between PM_2.5_ exposure and DWI.

**Results:**

In a study population of 7602 children (mean [SD] age, 119.1 [7.42] months; 3955 [52.0%] female; 160 [ 21.%] Asian, 1025 [13.5%] Black, 1616 [21.3%] Hispanic, 4025 [52.9%] White, and 774 [10.2%] other [identified by parents as American Indian/Native American or Alaska Native; Native Hawaiian, Guamanian, Samoan, other Pacific Islander; Asian Indian, Chinese, Filipino, Japanese, Korean, Vietnamese, or other Asian; or other race]), associations were seen between annual ambient PM_2.5_ and hemispheric differences in white matter microstructure. Hemisphere-stratified models revealed significant associations between PM_2.5_ exposure and restricted isotropic intracellular diffusion in the left cingulum, in the left superior longitudinal fasciculus, and bilaterally in the fornix and uncinate fasciculus. In tracts with strong positive associations, a PM_2.5_ increase from 8 to 12 μg/m^3^ was associated with increases of 2.16% (95% CI, 0.49%-3.84%) in the left cingulum, 1.95% (95% CI, 0.43%-3.47%) in the left uncinate, and 1.68% (95% CI, 0.01%-3.34%) in the right uncinate. Widespread negative associations were observed between PM_2.5_ and mean diffusivity.

**Conclusions and Relevance:**

The findings of this cross-sectional study suggest that annual mean PM_2.5_ exposure during childhood is associated with increased restricted isotropic diffusion and decreased mean diffusivity of specific white matter tracts, potentially reflecting differences in the composition of white matter microarchitecture.

## Key Points

**Question:**

Is annual residential exposure to particulate matter 2.5 μm or less in diameter (PM_2.5_) associated with neuroimaging diffusion markers of white matter microstructure?

**Findings:**

This cross-sectional study of 7602 children 9 to 10 years of age found evidence of an association between PM_2.5_ exposure and hemispheric differences in white matter microstructure. In hemisphere-specific models, adjusted for confounding variables, statistically significant positive associations were observed between PM_2.5_ and restricted isotropic diffusion, and statistically significant negative associations were observed between PM_2.5_ and mean diffusivity.

**Meaning:**

Findings from this study suggest that exposure to PM_2.5_ may be associated with differences in white matter microarchitecture, supporting a need for further improvements in air quality to protect the developing brain.

## Teaser

This cross-sectional study of children 9 to 10 years of age assesses associations between exposure to annual ambient particulate matter 2.5 μm or less in diameter and white matter microarchitecture.

## Introduction

Ambient airborne particulate matter is composed of suspended particles with an aerodynamic diameter of 2.5 μm or less (PM_2.5_).^1^ Long-term exposure to PM_2.5_ is reportedly associated with adverse nervous system effects.^2^ Animal studies^3,4,5,6^ have indicated that inhaled PM_2.5_ leads to neuroinflammation and oxidative stress, which may induce neuronal injury and affect glial support cells. Recent magnetic resonance imaging (MRI) studies have suggested an association of PM_2.5_ exposure with brain structure and volume,^7,8^ including white matter,^9,10,11,12^ which is primarily made up of myelinated axons and glial support cells.^13^

The potential impingement on key neurodevelopmental processes by PM_2.5_ exposure may cause lifelong health effects.^14^ Myelination and improved microstructural organization of white matter pathways continue throughout childhood and into young adulthood, ultimately allowing for improved signal transduction and communication between distal brain regions among cognitive and emotional systems.^15,16,17^ Studies have found that air pollution is associated with smaller white matter surface area in children,^11^ increases in myo-inositol, a brain metabolite involved in cell membrane and myelination,^18^ and reduced fractional anisotropy (FA).^12,19^ These studies^11,12,18,19^ are primarily based on smaller, localized populations, and results may have limited generalizability. Furthermore, exposure levels in these populations average above the current US Environmental Protection Agency standard of 12 μg/m^3^.^3,9,20^ Further research is warranted to examine the potential effects of exposure to levels of PM_2.5_ at or below regulatory standards across larger, more geographically diverse populations of children and using more advanced diffusion weighted imaging (DWI) techniques.

**Figure 1.**
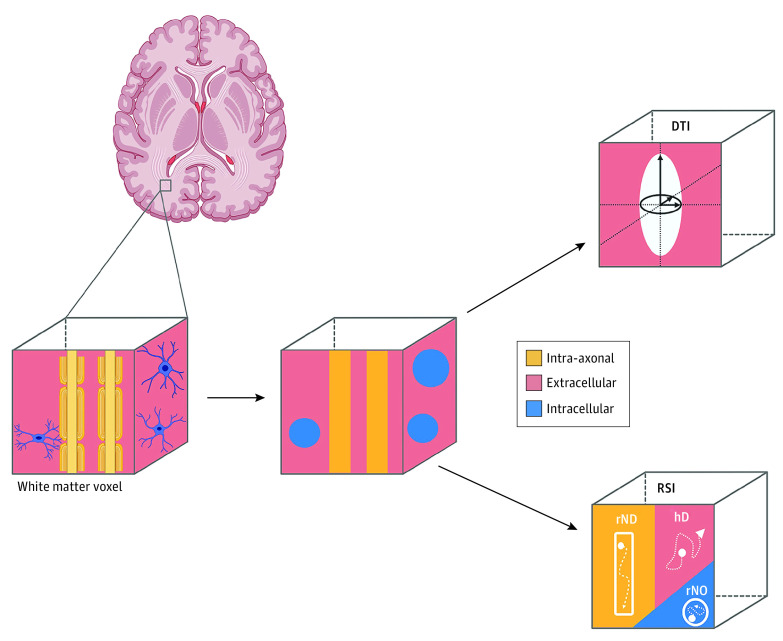
Diffusion-Weighted Imaging (DWI) Modeling Approaches Illustration of the biological components of white matter in an imaging voxel and schematic representation of the 2 diffusion tensor imaging (DTI) modeling approaches used in this study: DTI and restricted spectrum imaging (RSI). Diffusion tensor imaging measures extracellular water diffusion across a voxel. Primary DTI outcomes include fractional anisotropy (FA) and mean diffusivity (MD). Although the magnitude and direction of these outcomes allow for inferences to be made regarding axonal structure and integrity, DTI only allows for quantification of a single principal direction of diffusion and does not allow for characterization of the relative contribution of neurite orientations within a single voxel. In contrast, RSI is a biophysical model that allows for estimates of compartmentalized hindered and restricted water diffusion. Primary RSI outcomes include total hindered diffusion (hD) as well as restricted isotropic intracellular diffusion (rN0) and restricted directional intracellular diffusion (rND), which together provide greater insight into the biological properties of the microstructure of white matter tissue. The hindered compartment could encompass diffusion within intracellular spaces that allow for diffusion greater than the diffusion length scale (typically approximately 10 μm for human DTI). Given that rN0 and rND are normalized with respect to the hD compartments, changes in restricted compartments are relative to the other compartments. Previous studies using both common DTI and novel RSI metrics have shown similarities in directionality of rND and FA but opposite associations between rN0 and MD metrics in white matter during childhood.^21^ Created with BioRender.com.

By modeling multishell high-angular resolution DWI data with a novel framework called restriction spectrum imaging (RSI), we aimed to characterize associations between ambient PM_2.5_ exposure and white matter microarchitecture in children 9 to 10 years of age from the Adolescent Brain Cognitive Development (ABCD) Study. Restriction spectrum imaging adopts a biophysical model that goes beyond conventional diffusion tensor imaging (DTI) techniques to distinguish different types of microstructural tissue compartments, including restricted water bounded by cell membranes (intracellular) and hindered water primarily within the extracellular space, where glial cell bodies and other neural processes increase the tortuosity of diffusion (Figure 1).^22,23,24,25^ Restriction spectrum imaging improves sensitivity and specificity in understanding tissue damage^26^ and normative changes in microstructural development.^21,27^ Given that particle pollution has been linked with impaired myelination and alteration of glial cells,^28,29^ we hypothesized that higher levels of ambient PM_2.5_ exposure would be associated with decreased restricted directional intracellular diffusion (rND) (eg, organized myelination) and increased restricted isotropic intracellular diffusion (rN0) (eg, glia and cell bodies). Previous work^21^ suggests a moderately positive correspondence between FA and rND and a large correspondence (in opposite directions) between mean diffusivity (MD) and rN0 in age-related white matter changes in early adolescence; thus, we hypothesized that PM_2.5_ exposure may be associated with decreased FA and MD. We also examined whether these associations varied by hemisphere and sex, given previous evidence of hemispheric and sex differences in associations between PM_2.5_ exposure and health outcomes.^7,8,30,31,32,33,34^

## Methods

### Study Population

Data were obtained from baseline assessments (September 1, 2016, to October 15, 2018) of the ABCD Study (2020 National Institute of Mental Health Data Archive 3.0 data release), a cohort study of participants 9 to 10 years of age in the US.^35,36,37^ The ABCD Study implemented identical protocols for recruitment and neuroimaging of all participants at 21 study sites across the US.^35,38,39,40,41,42,43^ The primary inclusion criteria were age and English proficiency; exclusion criteria included severe sensory, intellectual, medical, or neurologic issues that would affect valid data collection (eMethods in the Supplement). Data analysis was performed from September 15, 2020, to June 30, 2021. Study sites obtained approval from their local institutional review boards, and centralized institutional review board approval was obtained from the University of California, San Diego. All parents or caregivers provided written informed consent; each child provided written assent. All data were deidentified before use. This study followed the Strengthening the Reporting of Observational Studies in Epidemiology (STROBE) reporting guideline.^44^

We further excluded participants with nonvalid addresses, low-quality or missing MRI, and incidental abnormal MRI findings (eMethods in the Supplement). Within-family nonindependence was managed by randomly including 1 sibling per family (eFigure 1 in the Supplement). The final analytic sample included 7602 participants (Table; eFigure 1 in the Supplement).

**Table.** Demographic Characteristics of the Final Study Data Set Compared With the Full Baseline ABCD Study Data Set^a^

Characteristic	Final data set (n = 7602)	Full data set (n = 11 884)	*P* value^b^
Age, mean (SD) [range], mo	119.1 (7.42) [107-133]	119.0 (7.50) [107-133]	.30
Familial relationships			
Single	5866 (77.2)	7900 (66.5)	<.001
Sibling	810 (10.7)	1810 (15.2)	
Twin	916 (12)	2138 (18.0)	
Triplet	10 (0.1)	30 (0.25)	
Sex			
Male	3955 (52.0)	6196 (52.2)	.85
Female	3647 (48.0)	5682 (47.8)	
Race and ethnicity			
Asian	160 (2.1)	252 (2.12)	.03
Black	1025 (13.5)	1784 (15.1)	
Hispanic	1616 (21.3)	2411 (20.3)	
White	4025 (52.9)	6182 (52.1)	
Other^c^	774 (10.2)	1247 (10.5)	
Educational level			
Less than HS diploma	358 (4.7)	593 (5.0)	.25
HS diploma or GED	676 (8.9)	1132 (9.5)	
Some college	1937 (25.5)	3080 (26.0)	
Bachelor	1938 (25.5)	3015 (25.4)	
Postgraduate	2685 (35.4)	4044 (34.1)	
Family income, $			
<50 000	1976 (26.0)	3224 (27.1)	.29
≥50 000 to <100 000	1987 (26.1)	3071 (25.9)	
≥100 000	2998 (39.4)	4565 (38.4)	
Don’t know or refuse	641 (8.4)	1016 (8.6)	
Parents employment status			
Working	5315 (70.2)	8218 (69.5)	.48
Unemployed	407 (5.4)	674 (5.7)	
Other	1847 (24.4)	2930 (24.8)	
Handedness			
Left	527 (6.9)	848 (7.1)	.37
Right	6097 (80.2)	9429 (79.4)	
Ambidextrous	978 (12.9)	1601 (13.5)	
MRI manufacturer			
GE Medical Systems	1795 (24.0)	2974 (25.7)	<.001
Philips Medical Systems	844 (11.3)	1516 (13.1)	
Siemens	4839 (64.7)	7100 (61.3)	
Perceived neighborhood safety, mean (SD) [range]	3.9 (0.97) [1.0-5.0]	3.9 (0.98) [1.0-5.0]	.90
Annual PM_2.5_, mean (SD) [range], μg/m^3^	7.66 (1.56) [1.72-15.90]	7.66 (1.56) [1.72-15.90]	.83
Motion [frame displacement], mean (SD) [range], mm	1.26 (0.26) [0.55-2.00]	1.39 (0.58) [0.55-16.14]	<.001
Date range of MRI	09/01/2016-10/15/2018	09/01/2016-10/15/2018	NA

Abbreviations: ABCD, Adolescent Brain Cognitive Development; GED, General Educational Development; HS, high school; MRI, magnetic resonance imaging; NA, not applicable.

^a^ Data are expressed as number (percentage) of participants unless otherwise indicated.

^b^ *P* value from the Pearson χ^2^ test comparing the distributions of categorical variables between the full ABCD Study baseline data set and the final analytic data set or *P* value from the analysis of variance test comparing means of continuous variables between the full ABCD Study baseline data set and the final analytic data set.

^c^ The “other” race and ethnicity category includes participants who were identified by their parents as American Indian/Native American or Alaska Native; Asian Indian, Chinese, Filipino, Japanese, Korean, Vietnamese, or other Asian; Native Hawaiian, Guamanian, Samoan, or other Pacific Islander; or other race.

### Estimation of PM_2.5_ Exposure

The methods used to estimate residential PM_2.5_ exposure have been previously described.^7^ Daily estimates of hybrid spatiotemporal PM_2.5_ models were used to aggregate daily PM_2.5_ exposure estimates across the 2016 calendar year at a resolution of 1 km^2^. These annual mean values were assigned to participants’ addresses at the time of their baseline visits. The mean (SD) annual PM_2.5_ concentration across all sites was 7.66 (1.56) μg/m^3^ (range, 1.72-15.90 μg/m^3^) (eFigure 2 in the Supplement).

### MRI Acquisition and Processing

The MRI data collection was harmonized across the 21 sites using 3 T scanners (Siemens Prisma, General Electric 750, Philips).^39,45^ The diffusion-weighted acquisition was conducted as previously described.^45^ After preprocessing of DWIs (eMethods in the Supplement), RSI was used to fit fiber orientation density functions to model rND, rN0, and total hindered diffusion (hD) (eg, primarily extracellular space around neurites).^45^ The DTI outcomes included FA and MD. Major white matter tracts were labeled with AtlasTrack using prior probabilities and orientation of long-range projection fibers.^46^ Probability estimates for each white matter tract were used to calculate weighted means of the RSI and DTI measures for all white matter fibers as well as key association, commissural, and projection fiber tracts,^45^ including the anterior thalamic radiations (ATR), cingulum in the cingulate gyrus (CGC), cingulum adjoining the hippocampus (CGH), corpus callosum (CC), corticospinal tract (CST), fornix (FX), uncinate fasciculus (UNC), inferior frontal occipital (IFO), inferior longitudinal fasciculus (ILF), and the superior longitudinal fasciculus (SLF).

### Sensitivity Analysis

We conducted sensitivity analyses adjusting models for population density and proximity to major roadways. We also evaluated the addition of random slopes by ABCD Study site to investigate geographic variability in the associations between PM_2.5_ exposure and DWI outcomes. Finally, we tested a possible interaction between PM_2.5_ exposure and assigned sex at birth.

### Statistical Analysis

We used hierarchical mixed-effects models with random intercepts by study site. Natural cubic splines for PM_2.5_ were fit with 2 knots at 7.05 and 8.31 μg/m^3^ derived from tertiles of exposure. We used an interaction term of PM_2.5_ × hemisphere and then fit hemisphere-stratified models (eMethods in the Supplement). All models were adjusted for covariates selected based on a directed acyclic graph, including sex, child’s age, parent-declared race and ethnicity, highest educational level of any household member, household income, parental employment status, a mean score of a 3-item assessment of parent perspectives of neighborhood safety, imaging device manufacturer, handedness, and motion artifact indexed by framewise displacement (eFigure 3 and eTable 3 in the Supplement). Analyses were performed using R, version 4.0.2 (R Foundation for Statistical Computing). An α = .05 was chosen, a priori, before any models were fit or analyzed as a threshold for significance. All reported *P* values are 1-sided.

## Results

This cross-sectional study of 7602 children (mean [SD] age, 119.1 [7.42] months; 3955 [52.0%] female; 160 [ 21.%] Asian, 1025 [13.5%] Black, 1616 [21.3%] Hispanic, 4025 [52.9%] White, and 774 [10.2%] other [identified by parents as American Indian/Native American, Alaska Native, Native Hawaiian, Guamanian, Samoan, other Pacific Islander, Asian Indian, Chinese, Filipino, Japanese, Korean, Vietnamese, other Asian, or other race]) suggests that significant associations, moderated by hemisphere, exist between annual mean ambient PM_2.5_ exposure and 2 measures of white matter microarchitecture.

### RSI-Derived White Matter Microstructure

**Figure 2.**
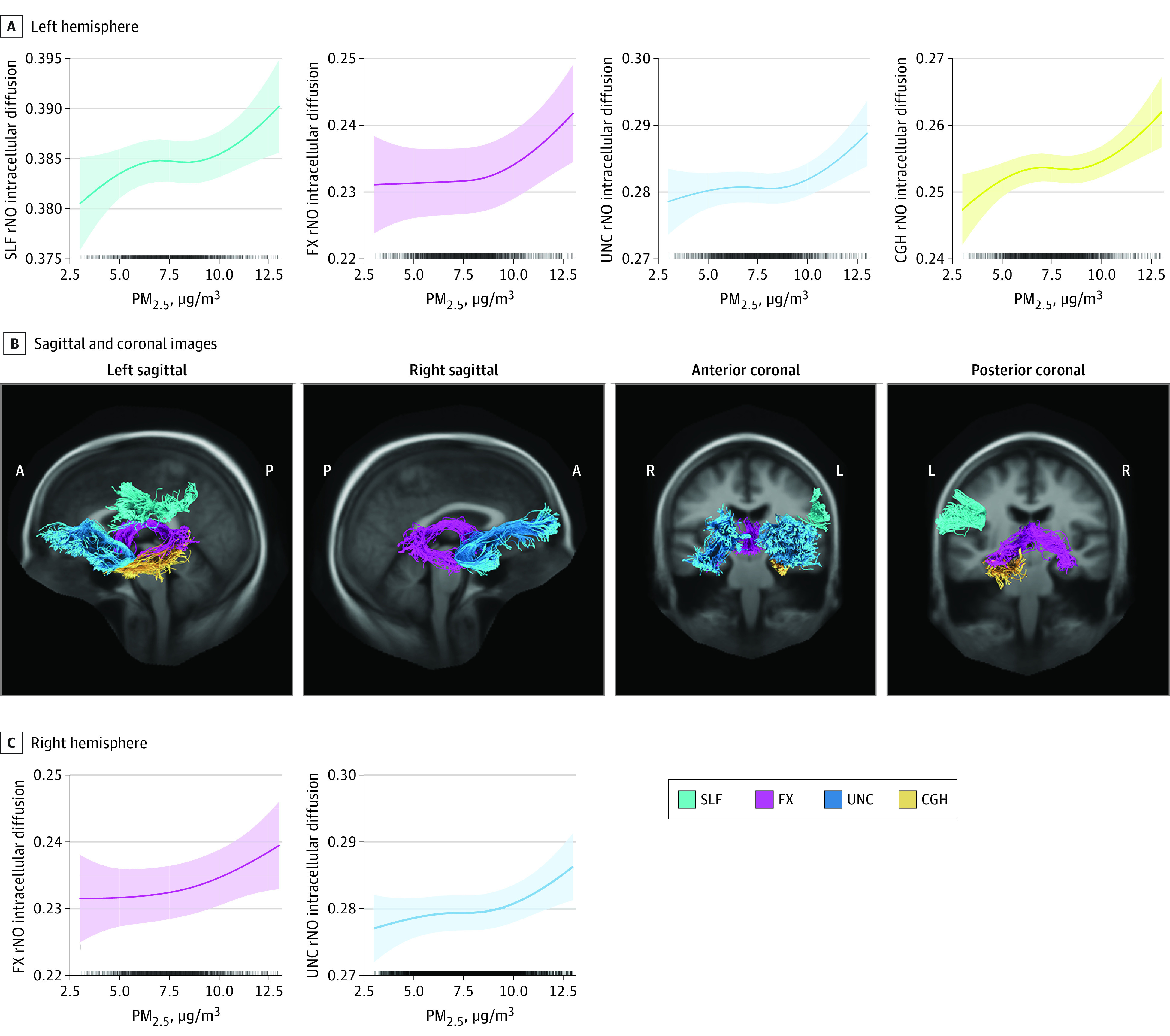
Associations Between Particulate Matter 2.5 μm or Less in Diameter (PM_2.5_) Exposure and Restricted Isotropic Diffusion Annual mean PM_2.5_ exposure is associated with increases in restricted isotropic intracellular diffusion (rN0) microarchitecture in specific white matter tracts of the left or right hemisphere. Spline plots reflect model-predicted values of rN0 associated with annual mean PM_2.5_ exposure, with all other model covariates held constant. Sagittal and coronal images of relevant white matter tracts are provided for reference and colored to match spline plots. A indicates anterior; CGH, cingulum hippocampal portion; FX, fornix; L, left hemisphere; P, posterior; R, right hemisphere; SLF, superior longitudinal fasciculus; and UNC, uncinate fasciculus.

Significant PM_2.5_ × hemisphere interactions were observed for the association of PM_2.5_ and all 3 RSI-derived measures for multiple tracts and the all white matter fiber summary (rN0: marginal *R*^2^ = 0.11; conditional *R*^2^ = 0.96; *P* < .001; rND: marginal *R*^2^ = 0.47; conditional *R*^2^ = 0.97; *P* < .001; hD: marginal *R*^2^ = 0.27; conditional *R*^2^ = 0.97; *P* < .001) (eTable 4 in the Supplement).^47^ Hemisphere-stratified post hoc models revealed significant, nonlinear, positive associations between PM_2.5_ and rN0 in the left CGH (marginal *R*^2^ = 0.16; conditional *R*^2^ = 0.17; *P* = .002), UNC (marginal *R*^2^ = 0.06; conditional *R*^2^ = 0.10; *P* = .006), and FX (marginal *R*^2^ = 0.14; conditional *R*^2^ = 0.28; *P* = .006) and significant linear positive associations between PM_2.5_ and rN0 in the right UNC (marginal *R*^2^ = 0.06; conditional *R*^2^ = 0.13; *P* = .02), the right FX(marginal *R*^2^ = 0.16; conditional *R*^2^ = 0.26; *P* = .04), and the left SLF (marginal *R*^2^ = 0.11; conditional *R*^2^ = 0.14; *P* = .03) (Figure 2). Hemisphere-stratified models did not reveal associations between PM_2.5_ and rND (marginal *R*^2^ range, 0.16-0.48; conditional *R*^2^ range, 0.21-0.51; *P* = .14-.98) and hD (marginal *R*^2^ range, 0.08-0.34; conditional *R*^2^ range, 0.13-0.37; *P* = .09-.95).

All models used the full exposure distribution of PM_2.5_ (1.72-15.90 μg/m^3^). For interpretability and quantification of associations, percent changes in outcomes associated with 4-μg/m^3^ increments of PM_2.5_ exposure were calculated using model-estimated predictions and SEs (eMethods in the Supplement). An increase in PM_2.5_ exposure from 4 to 8 μg/m^3^ was associated with percent increases that ranged from 0.25% (95% CI, −3.08% to 3.58%) to 1.44% (95% CI, −0.22% to 3.10%) in the 6 tracts evaluated (plus the all fiber summary), whereas an increase in PM_2.5_ exposure from 8 to 12 μg/m^3^ was associated with larger rN0 percent increases that ranged from 0.93% (95% CI, −0.10% to 1.97%) to 3.01% (95% CI, −0.39% to 6.40%) (eTable 5 in the Supplement). In tracts with strong positive associations, a PM_2.5_ increase from 8 to 12 μg/m^3^ was associated with increases of 2.16% (95% CI, 0.49%-3.84%) in the left cingulum, 1.95% (95% CI, 0.43%-3.47%) in the left uncinate, and 1.68% (95% CI, 0.01%-3.34%) in the right uncinate. Percent changes in rN0 according to household income and 6-month increases in age were estimated to contextualize the observed air pollution associations. In all models, age was included as a continuous variable with 1-month units, but for percent change calculations, 6-month increments were chosen as a reasonable time frame to capture developmental changes in white matter microstructure, given the 2-year age range of the study population. Increases in household income categories were not associated with rN0; percent changes ranged from −0.76% (95% CI, −2.4% to 0.61%) to 0.20% (95% CI, −1.17% to 1.60%); a 6-month increase in age was associated with percent changes in rN0 that ranged from 0.57% (95% CI, −2.44% to 3.58%) to 1.26% (95% CI, 0.11 to 2.41%) (eTable 5 in the Supplement).

### DTI-Derived White Matter Microstructure

**Figure 3.**
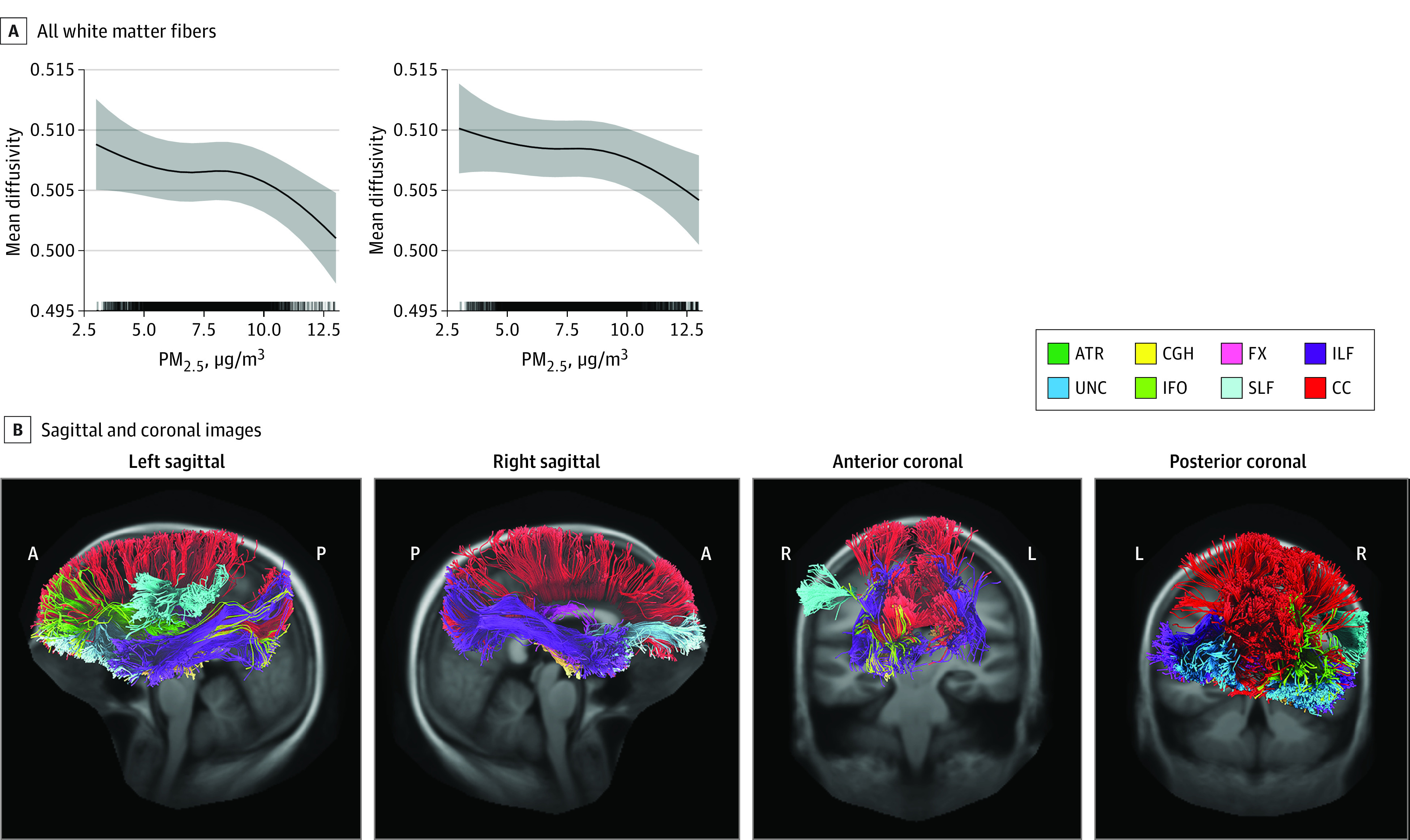
Associations Between Particulate Matter 2.5 μm or Less in Diameter (PM_2.5_) Exposure and Mean Diffusivity (MD) Annual mean PM_2.5_ exposure is associated with decreases in MD in all white matter fibers and 8 tracts of interest. Sagittal and coronal illustrations of relevant white matter tracts are provided for reference and colored to match spline plots (for details, see eFigure 4 in the Supplement). A indicates anterior; ATR, anterior thalamic radiations; CC, corpus callosum; CGH, cingulum hippocampal portion; FX, fornix; IFO, inferior fronto-occipital; ILF, inferior longitudinal fasciculus; L, left hemisphere; P, posterior; R, right hemisphere; SLF, superior longitudinal fasciculus; and UNC, uncinate.

For mean MD and FA, significant PM_2.5_ × hemisphere interactions were observed for all tract estimates and the all white matter fiber summary (FA: marginal *R*^2^ = 0.64; conditional *R*^2^ = 0.98; *P* < .001; MD: marginal *R*^2^ = 0.52; conditional *R*^2^ = 0.98; *P* < .001) (eTable 6 in the Supplement). Hemisphere-stratified models did not reveal significant associations between PM_2.5_ and FA (marginal *R*^2^ range, 0.32-0.61; conditional *R*^2^ range, 0.37-0.68; *P* = .09-.82). Models revealed significant, nonlinear, negative associations between PM_2.5_ and MD in the left hemisphere all white matter fiber summary estimate (marginal *R*^2^ = 0.51; conditional *R*^2^ = 0.58; *P* = .003); the left ATR (marginal *R*^2^ = 0.52; conditional *R*^2^ = 0.61; *P* = .004), CGH (marginal *R*^2^ = 0.62; conditional *R*^2^ = 0.65; *P* < .001), FX (marginal *R*^2^ = 0.62; conditional *R*^2^ = 0.71; *P* < .001), SLF (marginal *R*^2^ = 0.21; conditional *R*^2^ = 0.24; *P* = .009), and UNC (marginal *R*^2^ = 0.36; conditional *R*^2^ = 0.41; *P* = .001); and the right ILF (marginal *R*^2^ = 0.30; conditional *R*^2^ = 0.33; *P* = .02), and UNC (marginal *R*^2^ = 0.43; conditional *R*^2^ = 0.50; *P* = .008). Linear negative associations were observed between PM_2.5_ and MD in the right hemisphere all white matter fiber summary estimate (marginal *R*^2^ = 0.51; conditional *R*^2^ = 0.57; *P* = .04), the left IFO (marginal *R*^2^ = 0.47; conditional *R*^2^ = 0.54; *P* = .02) and ILF (marginal *R*^2^ = 0.28; conditional *R*^2^ = 0.30; *P* = .02), and the right CGH (marginal *R*^2^ = 0.64; conditional *R*^2^ = 0.67; *P* = .046) and FX (marginal *R*^2^ = 0.63; conditional *R*^2^ = 0.71; *P* = .01) (Figure 3, eFigure 4 in the Supplement).

Similar to rN0, more pronounced negative associations were observed with an exposure increase from 8 to 12 μg/m^3^, ranging from −1.06% (95% CI, −1.85% to −0.27%) to −0.56% (95% CI, −1.31% to 0.18%) compared with percent changes associated with lower levels exposure (ranging from −0.43% [95% CI, −1.13% to 0.27%] to −0.01% [95% CI, −0.77% to 0.74%]) (eTable 7 in the Supplement). A 6-month increase in age was associated with decreasing MD, ranging from −0.53% (95% CI, −1.02% to −0.03%) to −0.23% (95% CI, −1.31% to 0.84%). Increases in household income were not associated with MD; changes ranged from −0.13% (95% CI, −0.88% to 0.63%) to 0.30% (95% CI, −0.46% to 1.06%) (eTable 7 in the Supplement).

### Sensitivity Analysis

The addition of 1 or both of our sensitivity covariates (distance to major roadways and population density) did not improve model fit or change associations. We did not see any meaningful variability in the random slopes across ABCD Study sites. In addition, no associations with sex were found for any outcomes.

## Discussion

This cross-sectional analysis used data from a diverse cohort of 7602 children 9 to 10 years of age located at 21 geographically diverse locations across the US. Our objective was to characterize associations between annual ambient PM_2.5_ exposure and white matter microarchitecture. We found evidence of an interaction with hemisphere for nearly every white matter tract analyzed. In hemisphere-stratified models, higher PM_2.5_ exposure was associated with increased rN0 in 2 tracts in the left hemisphere only and bilaterally in 2 tracts. Higher exposure was associated with decreases in MD in 3 tracts in the left hemisphere only, bilaterally in 4 tracts, and in the corpus callosum. Significant hemisphere-specific associations were not observed between PM_2.5_ and rND, hD, or FA. These findings suggest that higher PM_2.5_ exposure is linked to increases in cellular barriers in white matter (reflected by decreases in MD), including increases in the isotropic compartment (reflected by increases in rN0), which may indicate changes to the cellular composition of key white matter tracts. To our knowledge, this is the first study to investigate how PM_2.5_ is associated with RSI-measured restricted and hindered water diffusion. The robustness of these findings is supported by strict quality control criteria for MRI inclusion, selection of covariates to control confounding, and sensitivity analyses (eMethods in the Supplement). Although the observed associations are small, it is plausible that repeated daily exposure to ambient PM_2.5_ across adolescence may have important implications for long-term neurophysiologic health outcomes of today’s youth.^48^

**Figure 4.**
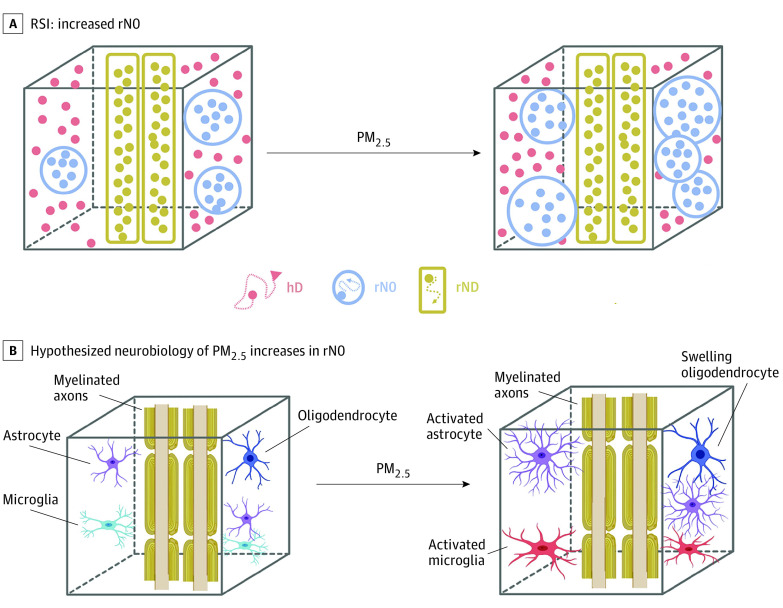
Hypothesized Neurobiological Mechanisms Associated With Increased Restricted Isotropic Diffusion Hypothesized neurobiological underpinning of particulate matter 2.5 μm or less in diameter (PM_2.5_)–associated increase in restricted isotropic intracellular diffusion (rN0). Given that rN0 represents diffusion within cells, it is hypothesized that increases of rN0 in white matter at 9 to 10 years may reflect an increase in the size or number of support and glial cells in response to exposure to PM_2.5_. An overall increase or change in cell numbers within a given white matter region could also contribute to overall decreases in mean diffusivity as measured by diffusion tensor imaging. hD indicates total hindered diffusion; rND, restricted directional intracellular diffusion; RSI, restriction spectrum imaging.

Our findings suggest that PM_2.5_ exposure may be associated with changes in intracellular microarchitecture of frontoparietal and limbic white matter circuitry, important for attention (SLF), emotional processing (UNC, CGH, and SLF), and memory (FX and SLF).^49,50,51,52^ Particle pollution may pass from the lungs into the bloodstream to infiltrate the blood brain barrier or create systematic secondary effects through inflammation.^4^ Microglia, the resident immune cells of the central nervous system, respond to pollutants and cause inflammatory activation.^28^ Activated microglia in animal and cell models exhibit increases in somal size compared with resting microglia.^53^ The rN0 reflects diffusion bound within cell membranes in spherical structures less than approximately 10 μm or within multiple cylindrical structures oriented such that diffusion is occurring equally in all directions. Therefore, our findings of increased rN0 may reflect differences in the number or size of glial cells (eg, oligodendrocytes, oligodendrocyte precursor cells, astrocytes, and/or microglia) in white matter tracts (Figure 4).^13^ Because increases in the restricted signal fraction along white matter tracts have been associated with normative development in the ABCD Study cohort, it is unclear to what extent these microstructural associations with PM_2.5_ reflect an acceleration of developmental processes vs an inflammatory response.^21^ A clinical study^54^ examining rN0 in patients with Parkinson disease found a 9.09% increase in rN0 in the bilateral thalamus of patients compared with healthy controls, pointing to the possibility that rN0 changes may reflect mechanisms underlying symptom origins. Further experimental animal studies using both cellular and neuroimaging techniques are warranted to better understand the implications of white matter microstructural changes.

Previous research^21^ has found that rND and FA tend to show similar patterns, whereas rN0 and MD are inversely associated in white matter tracts during early adolescence. Given these known patterns, the negative associations between PM_2.5_ and MD observed here suggest an increased barrier to water diffusion, which is congruent with potential increases in the number or size of support cells. Given that MD quantifies the magnitude of diffusion, whereas FA depends on the overall directionality of diffusion within a voxel,^55^ our findings suggest that PM_2.5_ may be increasing the number of cellular boundaries but not changing cellular processes that contribute to unidirectional water diffusion, such as axonal organization and/or myelination. Moreover, given the known associations between FA and rND metrics, a lack of association between PM_2.5_ and these 2 markers corroborates this conclusion.

Despite congruent findings between DTI and RSI outcomes, the notable negative associations between PM_2.5_ and MD in the current study contrast with a previous positive association noted in a cohort of children 9 to 10 years of age from Rotterdam, the Netherlands.^9^ PM_2.5_ composition varies by geographic location, and previous evidence^2,56^ suggests that certain PM_2.5_ components are differentially detrimental to health, which may explain these contrasting findings. Participants from the previous study^9^ were also exposed to much higher overall levels of PM_2.5_ exposure (mean, 16.5 μg/m^3^), which may also contribute to this discrepancy because higher levels of exposure may lead to more severe cellular or myelin disruption (reflected by increases in MD). Finally, the current study and the study by Lubczyńska et al^9^ used different diffusion-MRI acquisition parameters; the previous study^9^ used a single shell DTI sequence, whereas the current study used a multishell high-angular resolution DWI sequence with various b values, allowing for increased sensitivity and specificity.^21,57^

Although the exact mechanisms underlying central nervous system asymmetries remain unknown,^58^ structural and functional differences have been noted between the 2 cerebral hemispheres at the macroscopic, microstructural, and molecular levels.^59^ A previous study^58^ of asymmetry in neurologic disorders suggests that typical asymmetries develop between the hemispheres, which may ultimately result in greater hemispheric differences in vulnerability to brain pathologic conditions. A previous study^58^ suggests that brain asymmetries occur via differences in functional genetic pathways of microtubule regulation, neurogenesis, and axonogenesis, which are involved in neuronal development and organization and the manifestation of hemispheric differences in gene expression. Thus, these asymmetries in brain structure and function may contribute to the hemisphere-specific patterns observed in this study.

### Limitations

This study has limitations. Because of limits in the ABCD Study air pollution data available in the 3.0 release, participants experienced varying time lags between their air pollution exposure estimation (2016) and MRI at the baseline study visit (2016-2018); an assumption was therefore made that the spatial distribution of air pollution estimates remained stable during this 2-year period. This assumption is supported by previous research that indicates that the spatial distribution of estimates of annual mean air pollution concentrations (using the current estimation methods) remained relatively stable in the US between 2008 and 2016.^60^ Future data releases from the ABCD Study are expected to contain full lifetime histories of air pollution exposure, which will allow for more temporal precision in cross-sectional analyses and longitudinal investigations.

Ambient outdoor PM_2.5_ exposure at a primary residence does not provide a full picture of a child’s yearly air pollution exposure. Data on indoor air pollution, school air pollution, and time at the residence, although not currently available, would further clarify how PM_2.5_ exposure is associated with white matter connectivity. Similarly, despite efforts to account for confounding in our analyses, it is possible that unmeasured confounders and residual confounding have introduced biases in the associations reported here.

In addition, this study was limited to PM_2.5_ exposure, yet other types of air pollution, including nitric dioxide, may affect the morphological features and development of children’s brains and their mental health.^9,19,61^ In previous studies^9,19^ with multipollutant analyses, single pollutant associations tended to become weakened by the addition of 1 of more pollutants; thus, the possibility exists that the PM_2.5_ associations reported here are somewhat biased and inflated by the inclusion of only a single air pollutant in our models. When data become available for the ABCD Study population, future analyses are planned to elucidate potential associations among ambient nitric dioxide_,_ ozone_,_ PM_2.5_ components, and DWI outcomes.

## Conclusions

To our knowledge, this was the first multisite US study to find associations between annual PM_2.5_ exposure and white matter microarchitecture. Most of the study population experienced PM_2.5_ exposure at or below 12 μg/m^3^, which is within US Environmental Protection Agency standards. These findings have important public health implications, given the ubiquity of PM_2.5_ exposure and its potential effects on white matter connectivity in children across the US.

## Notes

Supplement. **eMethods.** Supplemental Methods **eFigure 1.** Flowchart of Final Sample **eFigure 2.** Distribution of PM_2.5_ Annual Average Exposure by ABCD Site **eFigure 3.** Directed Acyclic Graph **eFigure 4.** Associations Between PM_2.5_ Exposure and Mean Diffusivity **eTable 1.** Description of Covariates Used in Statistical Analyses **eTable 2.** Comparison of Population Characteristics Across Data sets **eTable 3.** Distributions of Population Characteristics at Baseline in Relation to Annual Average PM_2.5_ **eTable 4.** PM_2.5_-by-Hemisphere Analyses for RSI Outcomes **eTable 5.** Percent Change in RSI rN0 Across Values of PM_2.5_ and Sociodemographic Characteristics for Significant Hemisphere-Specific Models **eTable 6.** PM_2.5_-by-Hemisphere Analyses for DTI Outcomes **eTable 7.** Percent Change in DTI MD Across Values of PM_2.5_ and Sociodemographic Characteristics for Significant Hemisphere-Specific Models
