# Supplementary material for: Association of Outdoor Ambient Fine Particulate Matter With Intracellular White Matter Microstructural Properties Among Children
Source: JAMA Netw Open. 2021 Dec 9;4(12):e2138300. doi: 10.1001/jamanetworkopen.2021.38300 (PMC8662373; doi:10.1001/jamanetworkopen.2021.38300)
Supplement: Supplement. — eMethods. Supplemental Methods eFigure 1. Flowchart of Final Sample eFigure 2. Distribution of PM2.5 Annual Average Exposure by ABCD Site eFigure 3. Directed Acyclic Graph eFigure 4. Associations Between PM2.5 Exposure and Mean Diffusivity eTable 1. Description of Covariates Used in Statistical Analyses eTable 2. Comparison of Population Characteristics Across Data sets eTable 3. Distributions of Population Characteristics at Baseline in Relation to Annual Average PM2.5 eTable 4. PM2.5-by-Hemisphere Analyses for RSI Outcomes eTable 5. Percent Change in RSI rN0 Across Values of PM2.5 and Sociodemographic Characteristics for Significant Hemisphere-Specific Models eTable 6. PM2.5-by-Hemisphere Analyses for DTI Outcomes eTable 7. Percent Change in DTI MD Across Values of PM2.5 and Sociodemographic Characteristics for Significant Hemisphere-Specific Models [file jamanetwopen-e2138300-s001.pdf]

Supplemental Online Content

Burnor E, Cserbik D, Cotter DL, et al. Association of outdoor ambient fine particulate matter with intracellular white matter microstructural properties among children. *JAMA Netw Open*. 2021;4(12):e2138300. doi:10.1001/jamanetworkopen.2021.38300

eMethods. Supplemental Methods

eFigure 1. Flowchart of Final Sample

eFigure 2. Distribution of $PM_{2.5}$ Annual Average Exposure by ABCD Site

eFigure 3. Directed Acyclic Graph

eFigure 4. Associations Between $PM_{2.5}$ Exposure and Mean Diffusivity

eTable 1. Description of Covariates Used in Statistical Analyses

eTable 2. Comparison of Population Characteristics Across Data Sets

eTable 3. Distributions of Population Characteristics at Baseline in Relation to Annual Average $PM_{2.5}$

eTable 4. $PM_{2.5}$-by-Hemisphere Analyses for RSI Outcomes

eTable 5. Percent Change in RSI rN0 Across Values of $PM_{2.5}$ and Sociodemographic Characteristics for Significant Hemisphere-Specific Models

eTable 6. $PM_{2.5}$-by-Hemisphere Analyses for DTI Outcomes

eTable 7. Percent Change in DTI MD Across Values of $PM_{2.5}$ and Sociodemographic Characteristics for Significant Hemisphere-Specific Models

This supplemental material has been provided by the authors to give readers additional information about their work.

© 2021 Burnor E et al. *JAMA Network Open.*

eMethods. Supplemental Methods

ABCD study design and sample characteristics: Sample recruitment at the ABCD study sites and schematic overview of the ACBD study has been reported previously in detail [1-7]. Participants were enrolled via school-based recruitment system in which any children at selected schools were invited to participate. Inclusion criteria for the ABCD study were: 1) age 9.00 to 10.99 years at the time of baseline assessment; 2) able to validly and safely complete the baseline visit including MRI; 3) Fluent in English. The ABCD study population is intended to represent a population-based, nonclinical sample; thus exclusion criteria included severe sensory, intellectual, medical, and neurological disorders (such as cerebral palsy, brain tumor, stroke, brain aneurysm, brain hemorrhage, subdural hematoma, multiple sclerosis, sickle cell disease, Lennox-Gastaut syndrome, Dravet syndrome, and Landau Kleffner syndrome), a history of traumatic brain injury, a diagnosis of schizophrenia, moderate or severe autism spectrum disorder, intellectual disability, gestational age less than 28 weeks and birthweight less than 1.2 kilograms, birth complications (besides those associated with prematurity) that resulted in hospitalization for more than a month, and a diagnosis of alcohol/substance abuse [8]. Additionally, exclusion criteria included conditions that would prevent children from completing ABCD study protocols, including MRI contraindications and non-correctable vision, hearing, or sensorimotor impairments [9].

Study sites obtained approval from their local institutional review boards (IRBs) and centralized IRB approval was obtained from the University of California, San Diego. All parents or caregivers provided written informed consent; each child provided written assent.

MRI scans performed during the baseline study visit were reviewed by neuroradiologists and classified according to the following categorical scoring system in order to identify and flag incidental findings: 0, image artefacts prevent radiology read; 1, no abnormal findings; 2, normal anatomic variant or common incidental finding, no referral necessary; 3, consider referral; 4, consider immediate referral [10]. During data cleaning for this analysis, only subjects with scans in categories 1 or 2 were retained, and subjects in categories 0, 3, or 4 were excluded. A previous study investigating the rates of incidental findings brain MRIs of the ABCD study cohort did not find systematic differences in the study population with respect to the MRI categorical scoring system [10]. Within the study dataset used in this analyses, a total of 2,412 subjects were removed on the bases of serious incidental MRI findings, poor quality MRI scans (see below), or missing MRI data.

© 2021 Burnor E et al. *JAMA Network Open.*

Further data cleaning involved removing subjects with nonvalid addresses (i.e. no estimate for average annual $PM_{2.5}$ exposure); we also randomly selected only one sibling from each family (eFigure 1). For most demographic variables, the children included in the final analytic sample were statistically similar to the overall ABCD population, with the exception of the 'race/ethnicity' variable (eTable1); children in the final sample were slightly more likely to be parent-identified as white or Hispanic and slightly less likely to be parent-identified as Black or Other ($p = 0.03$). The children in the final sample were also more likely to have been scanned in an MRI machine produced by Siemens, and less likely to have been scanned in a machine produced by GE Medical systems or Philips Medical Systems ($p < 0.001$). They also had lower overall frame displacement (mm) during MRI scanning ($p < 0.001$); this was to be expected, as removing subjects with high frame displacement was a step in the data cleaning process. For a full breakdown of subjects removed during data cleaning and a comparison between the baseline dataset and the final analytic dataset, see eTable 2.

Imaging pulse sequences, and image processing methods: T1-weighted anatomical acquisition was a 3D T1-weighted inversion prepared RF-spoiled gradient echo scan using prospective motion correction when possible, at the resolution of 1mm isotropic. The diffusion weighted acquisition utilized a multi-shell, multiband Echo Planar Imaging (EPI) with a slice acceleration factor of 3 at the resolution of 1.7mm isotropic and 96 gradient directions, including: 7 b0s and 4 b-values (6 directions with b=500 s/mm^2; 15 direction with b=1000 s/mm^2; 15 directions with b=2000 s/mm^2; and 60 directions with b=3000 s/mm^2).

Quality Control: Automated quality control procedures include the calculation of metrics such as signal-to-noise ratio (SNR) and head motion statistics. For DWI series, head motion was estimated by registering each frame to a corresponding image synthesized from a tensor fit, accounting for variation in image contrast across diffusion orientations. An overall head motion is quantified as the average of an estimated frame-to-frame displacement (FD; mm) from head motion. [11]. At the central ABCD Data Analysis, Informatics, and Resource Center (DAIRC), all images underwent quality control (QC) [12]. Trained technicians at the DAIRC then inspect images for quality control (see [12] for details). Only image types with motion < 2mm and images that passed a rigorous QC for all categories (i.e. imgincl_dmri_include = 1) were included in our final analyses. These QC categories include the following: the images had no serious MRI findings requiring clinical referral, the imaging series passed a raw quality control inspection, the total number of repetitions for all 'OK' scans was 103 or more, the T1 series passed a raw QC inspection, the B0

© 2021 Burnor E et al. *JAMA Network Open.*

unwarp data was available, the FreeSurfer QC did not fail, the imaging passed a manual post-processing QC test, the maximal dorsal cutoff score was less than 47, and that maximal ventral cutoff score was less than 54.

Preprocessing: Preprocessing of the diffusion weighted images included: eddy current correction [13]; head motion correction [11]; adjustment of gradients for head motion [11,14]; robust tensor fit to identify and exclude dark frames due to abrupt head motion[15]; B_0 distortion and gradient distortion correction using opposite phase encoding pairs of b=0s [16,17]; b0 registration to T1-weighted structural images using mutual information [18]; and cubic interpolation to resample at the 1.7 isotropic resolution.

Fiber tractography: Details regarding fiber tract segmentation have been previously published (see [12] for details). A probabilistic atlas-based method was utilized via AtlasTrack [11]. This fiber atlas contains prior probabilities and orientation information for specific projection fibers. For each subject, sMRI images were nonlinearly aligned to the atlas using discrete cosine transforms [19], and diffusion derived orientation were compared to the atlas fiber orientation in order to refine *a priori* tract locations to create individualized fiber tract regions of interest. Voxels primarily comprised on gray matter or cerebral spinal fluid as derived by FreeSurfer's automated brain segmentation [20], are excluded from analysis.

Restriction spectrum imaging (RSI): RSI modeling was implemented for separate fiber orientation density (FOD) functions to model, as fourth order spherical harmonic functions, two volume fractions including intracellular and extracellular diffusion within a single voxel [21-23]. Longitudinal diffusion parameter was held constant for both fractions at $1x10^{-3}mm^2/s$ and for the intracellular fraction the transverse diffusion parameter was modeled as 0, whereas the extracellular fraction set the transverse parameter to $0.9x10^{-3}mm^2/s$. Measures of interest derived from the RSI model included the directional (rND) and isotropic (rN0) intra-cellular diffusion (e.g. restricted water bounded by membrane of cells) and total hindered extra-cellular diffusion (hD; e.g. hindered space around the neurites) spaces [12]. Each of these measures is normalized and is defined as the Euclidean norm (square root of the sum of squares) for the corresponding model coefficients divided by the norm of all model coefficients. As such, these measures are unitless and range from 0 to 1 and the square of each measure is equivalent to the signal fraction for their respective model components. N0 is derived from the 0th order spherical harmonic coefficients of the restricted fraction and is the contribution of intracellular space to isotropic diffusion in a given voxel. ND is calculated from the 2nd order spherical harmonic coefficients of the restricted fraction and reflects oriented diffusion. ND is thought to be similar to FA except is less affected by crossing fivers. hD is calculated from the norm of the

© 2021 Burnor E et al. *JAMA Network Open.*

0th, 2nd, and 4th order coefficients of the hindered fractions, and reflects the overall contribution of diffusion from the extracellular space [12].

Diffusion tensor image (DTI): DTI outcomes were calculated using a standard, linear estimation approach [24] using the 6 directions at b=500 s/mm^2, 15 directions at b=1000 s/mm^2.

Covariates: A full description of each covariate used in statistical analyses can be found in eTable 1. The inclusion of each covariate was decided upon using a directed acyclic graph (DAG) [25] to identify confounders that may predict white matter development and exposure to ambient air pollutants (eFigure 3). Selection of potential confounders were based on both prior knowledge and empirical data [26]. Specifically, race and ethnicity and socioeconomic status were included in this analysis due to abundant previous evidence that large disparities in magnitude and severity of air pollution exposure exist along racial and socioeconomic lines [27-29]. As previously reported [9], distribution of annual $PM_{2.5}$ exposures are associated with both demographic and social covariates in the ABCD sample (see eTable 3). Therefore, all models in the main analysis were adjusted for sociodemographic covariates, including: a) child's sex, age and race/ethnicity; b) family socioeconomic status (SES): highest education (of any household member), total household income, parental employment status; c) neighborhood quality: average score of three-items assessing parent perspectives of neighborhood safety [30], and for MRI covariates, including an indicator of the imaging device manufacturer, subject's hand dominance, and average framewise displacement to account for MRI motion.

$PM_{2.5}$ exposure estimates capture both local and regional sources of air pollution, and the urban built environment is likely to impact $PM_{2.5}$ exposure. In sensitivity analyses, we assessed additional confounding effects of population density and distance to road. Specifically, residentially derived United Nations population density was measured as persons per km^2 (based on population counts of the 2010 national census tract adjusted for potential underreporting across the world) [31] as a proxy for urbanicity, and distance to major roads and highways in meters [32] was treated as a categorical variable reflecting those living <150, 150-300m, 300-600m, or > 600m based on previous studies showing that near-roadway pollutants decay to background levels by approximately 115-570m [33]. An exploratory analysis was also conducted to assess whether interaction exists between annual ambient $PM_{2.5}$ exposure and sex at birth.

© 2021 Burnor E et al. *JAMA Network Open.*

Statistical analysis:

Statistical models: Based on a directed acyclic graph (DAG) approach [25], we identified potential confounders of interest and then examined potential differences in socio-demographic factors across $PM_{2.5}$ quintiles using analysis of variance (ANOVA) for continuous variables and Pearson's Chi-square test for categorical variables. We employed hierarchical mixed effects models in R to analyze the associations between annual average residential $PM_{2.5}$ exposure and tract-specific RSI and DTI measures. An exploration of the shapes of the association highlighted potential non-linearity in most, but not all, of the associations; thus, we opted to use a natural spline function with two knots ($PM_{2.5}$ = 7.05 ug/m^3 and 8.31 ug/m^3) to allow for flexibility in the associations between annual $PM_{2.5}$ exposure and white matter microstructure. Knots were selected according to tertiles of the overall $PM_{2.5}$ exposure distribution. Knot selection was performed by fitting a series of models with increasing numbers of knots (starting with one), until we determined that model fit was not improved by the addition of new knots. Due to the large number of tracts and outcomes to evaluate, we chose to retain the same natural spline structure for all models – even when there was not strong evidence for non-linearity – in order to simplify interpretations of results and comparisons between outcomes and tracts. As previous studies have found hemispheric differences in brain imaging outcomes, we examined a cross-product term of $PM_{2.5}$ by hemisphere to assess whether an interaction between $PM_{2.5}$ exposure and hemisphere exists (Equ 1). We then examined the associations between $PM_{2.5}$ exposure and RSI and DTI measures in hemisphere-stratified models (Equ 2). For all models, we included a random intercept for ABCD sites (j) in order to account for between-site variability (Equ 1 and 2). For models with a cross-product term by hemisphere, we included a nested random effect for subject (i) in order to account for within-subject variability in MRI readings in two hemispheres (Equ 1). In a sensitivity analysis, we explored the possibility of geographic variability in the associations between $PM_{2.5}$ exposure and our outcomes by fitting Equ. 2 models with random slopes by site. All models were also adjusted for the sociodemographic covariates described above (denoted W_i) and the MRI covariates described above (denoted Z_i). In Equations 1 and 2, y_{ij} denotes the measured outcome of interest (e.g. DTI or RSI outcome) for participant i, from study site j at baseline (ages 9-10). x_i denotes a personalized summary of $PM_{2.5}$ exposure (e.g., assigned to residential address of the 9-10 year old participant i). S_m denotes the mth spline term and δ_m denote the parameter estimate for the S_m spline. M indicates the total number of spline terms in the model. Finally, random effects at the level of study site, and, in Equation 1, at the subject level, are denoted.

© 2021 Burnor E et al. *JAMA Network Open.*

$$Y_{ij} = \beta_0 + \sum_{m=1}^{M}[\delta_m S_m(x_j)] \times Hemisphere + \beta_1 W_i + \beta_2 Z_i + U_{[i]j} + \varepsilon_{ij} \quad \text{Equ. 1}$$

$$Y_{ij} = \beta_0 + \sum_{m=1}^{M}[\delta_m S_m(x_j)] + \beta_1 W_i + \beta_2 Z_i + U_j + \varepsilon_j \quad \text{Equ. 2}$$

Statistical Model Interpretation

Spline models do not allow for a straightforward interpretation of model coefficients. Therefore, in order to quantify the magnitude of associations between $PM_{2.5}$ exposure and RSI/DTI outcomes and to capture shifts in the direction and magnitude of associations across the range of $PM_{2.5}$ exposure, we calculated percent change in model-predicted values for RSI and DTI outcomes across increments in $PM_{2.5}$ exposure as follows. All spline models were fit to the entire analytic dataset, with $PM_{2.5}$ exposures ranging from 1.72 to 15.90 μg/m^3. Percent change estimates are only reported to allow for easier interpretation and quantification of associations. Model-predicted estimates, or marginal means, (denoted *E*) with standard errors for outcome variables were obtained at three levels of $PM_{2.5}$ exposure (4 μg/m^3, 8 μg/m^3, and 12 μg/m^3) with all other covariates held constant. 8 μg/m^3 was chosen as a central inflection point by visual inspection of our spline plots (Figures 2 and 3), most of which demonstrate a shift in the slope of association at around 8 μg/m^3. We opted to exclude model-predicted marginal means below 4 μg/m^3 and above 12 μg/m^3 from our percent change calculations due to the high uncertainty associated with these exposure levels; relatively few datapoints (less than 3% of the dataset) exist at these levels of exposure (n = 165). Percent change in outcome was then calculated across 4 μg increments of $PM_{2.5}$ exposure (Equ. 3). Percent change in DWI outcomes was also calculated across levels of two other model predictors: household income and across a 6-month increase in age. These were calculated as reference points to compare age- and sociodemographic-related differences in white matter outcomes with our main findings related to air pollution-associated differences in our outcomes. The standard errors for the percent change calculations (denoted *SE*) was estimated using the approach outlined in the U.S. Census Bureau's documentation for calculating percent change within the American Community Survey Data [34], using standard errors obtained from model-predicted estimates (Equ. 4).

$$Percent\ Change = \frac{E_{\text{upper}} - E_{\text{lower}}}{E_{\text{lower}}} * 100 \quad \text{Equ. 3}$$

$$SE = \left|\frac{E_{\text{upper}}}{E_{\text{lower}}}\right| * \sqrt{\frac{SE_{upper}^2}{E_{upper}^2} + \frac{SE_{lower}^2}{E_{lower}^2}} * 100 \quad \text{Equ. 4}$$

© 2021 Burnor E et al. *JAMA Network Open.*

eFigure 1. Flowchart of final sample

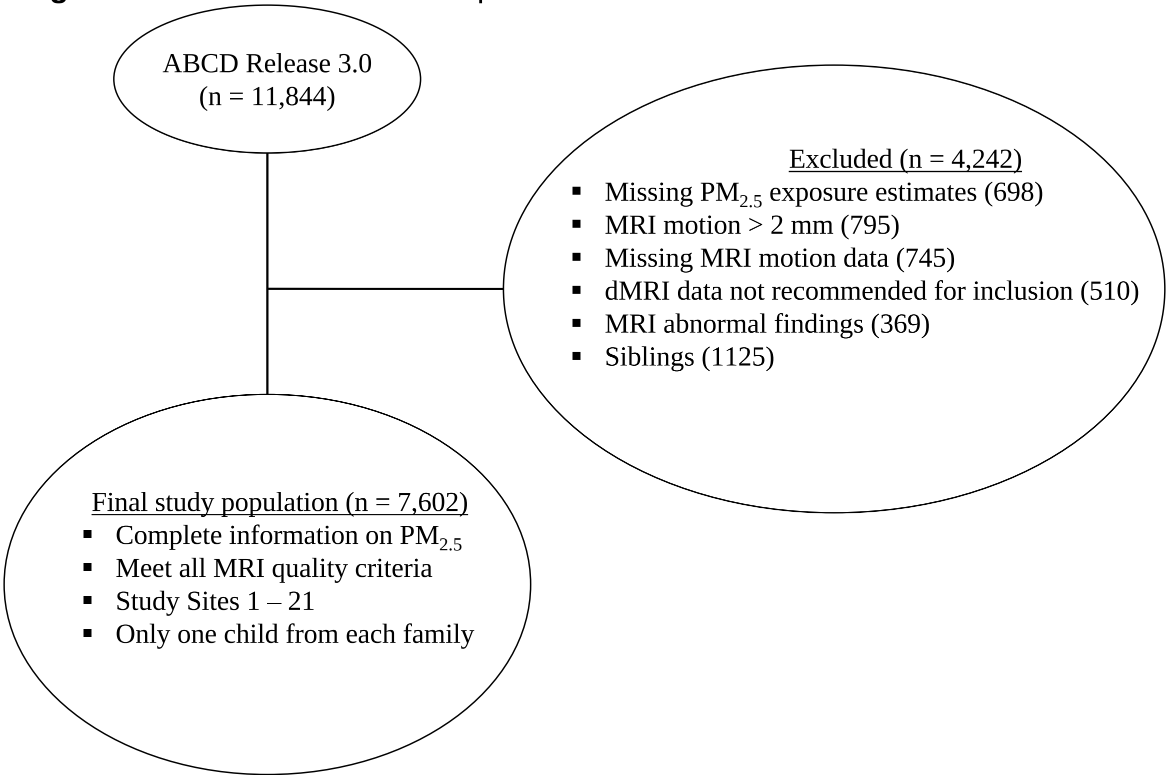

© 2021 Burnor E et al. *JAMA Network Open.*

eFigure 2. Distribution of $PM_{2.5}$ annual average exposure by ABCD site

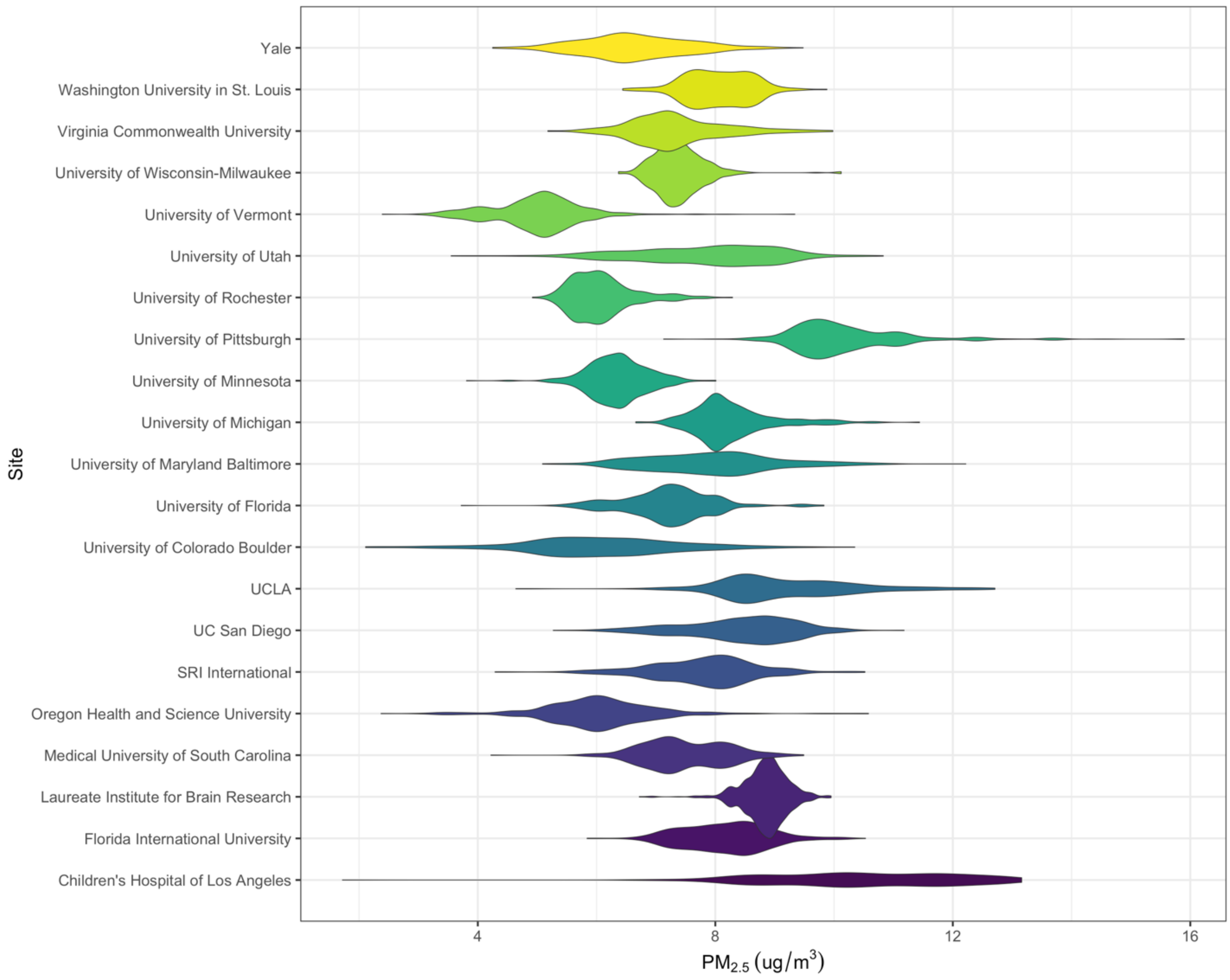

© 2021 Burnor E et al. *JAMA Network Open*.

eFigure 3. Directed acyclic graph

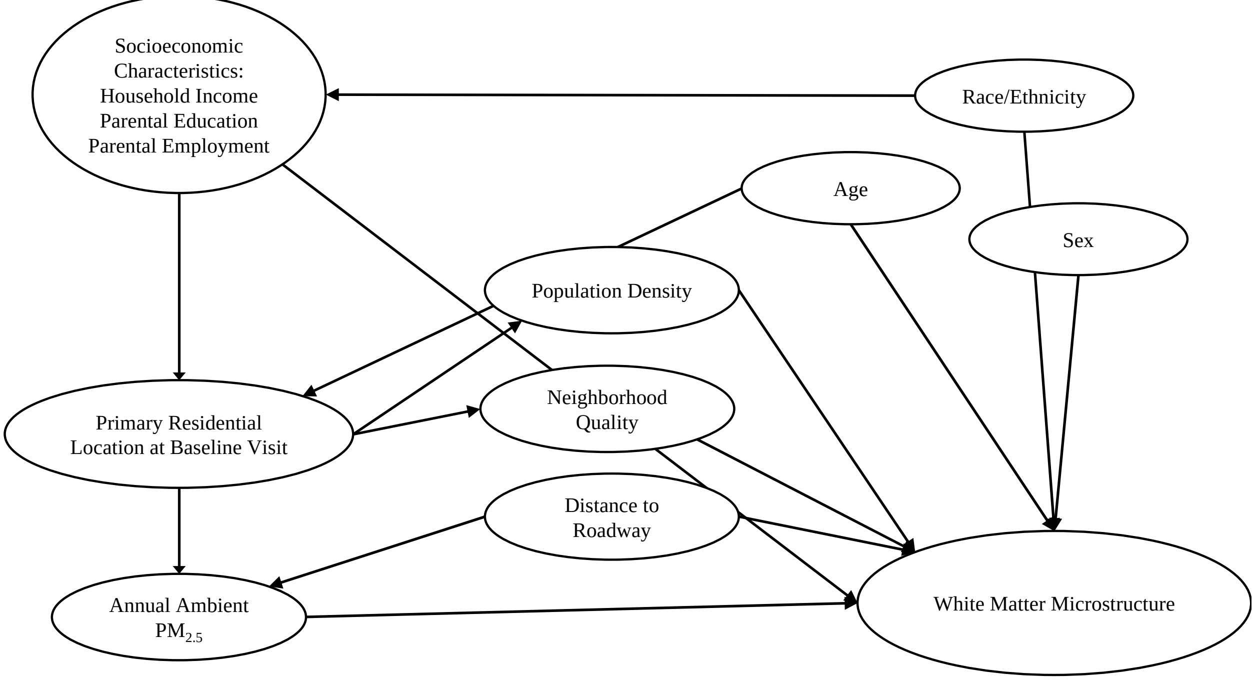

Directed Acyclic Graph of potential confounders that may predict white matter neurodevelopment and exposure to ambient air pollutants

© 2021 Burnor E et al. *JAMA Network Open*.

eFigure 4. Associations between PM2.5 Exposure and Mean Diffusivity

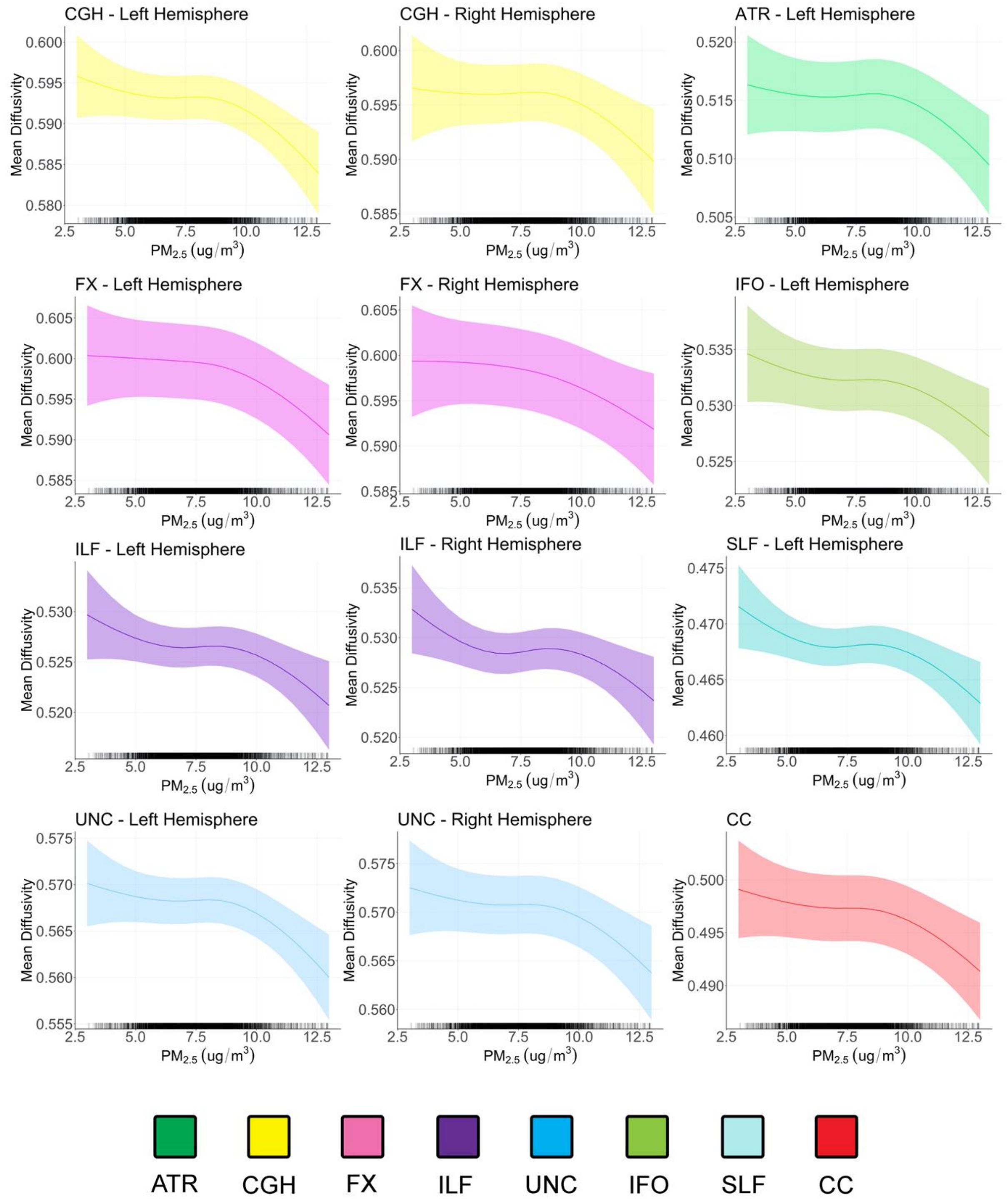

Annual average $PM_{2.5}$ exposure relates to decreases in mean diffusivity (MD) in 8 tracts of interest. Spline plots reflect model-predicted values of MD in relation to annual average $PM_{2.5}$ exposure, with all other model covariates held constant. Sagittal and coronal illustrations of relevant white matter tracts are provided in main Figure 3 for reference and colored to match spline plots. Abbreviations: ATR = anterior thalamic radiations (green); CC = corpus callosum (red); CGH = cingulum hippocampal portion (yellow); FX = fornix (magenta); IFO = inferior fronto-occipital (lime green); ILF = inferior longitudinal fasciculus (purple); UNC = uncinate (blue); SLF = superior longitudinal fasciculus (turquoise).

© 2021 Burnor E et al. *JAMA Network Open.*

eTable 1. Description of covariates used in statistical analyses

ABCD variable name	Description	Description of variable from NIMH*
(1) Sociodemographic		
abcd_site	Anonymized site name for the participant and visit.	-
interview_age	Age (in months)	Age is rounded to chronological month. If the research participant is 15-days-old at time of interview, the appropriate value would be 0 months.
sex_at_birth	Sex	Sex of the subject at birth.
race_ethnicity	Child's race/ethnicity	Dummy codes of race/ethnicity of the participant: 1 = White; 2 = Black; 3 = Hispanic; 4 = Asian; 5 = Other. The 'Other'
rel_family_id	Family ID	Family ID is unique to each family of the ABCD study, participants belonging to the same family share a family ID.
high.educ	Parental education (highest education of any member of household)	Highest education was defined as the highest education attained for reported education among caregivers. It was reported in incremental categories ranging from never educated/kindergarten through doctoral-level graduate degree. Dummy codes of parental highest education: 1 = less than High School Diploma; 2 = High School Diploma/GED; 3 = Some College; 4 = Bachelor; 5 = Post Graduate Degree. (DEAP variable)
overall.income	Family income (total combined).	Total combined family income for the past 12 months that includes income (before taxes and deductions) from all sources. Dummy codes of total combined family income: 1 = <50K; 2 = >=50K & <100K; 3 = >=100K. (DEAP variable)
demo_prnt_empl_p	ABCD Parent Demographics Survey: current employment status.	Categories of current employment status of parent have been recoded into the following dummy codes: 1 = Working now: FULL TIME/PART TIME; 2 = Stay at Home Parent; 3 = Unemployed; 4 = Other
(2) Neighborhood safety		

© 2021 Burnor E et al. *JAMA Network Open.*

ABCD variable name	Description	Description of variable from NIMH*
neighb_phenx_sum_p	Calculated average score derived from three questions of the ABCD Parent Neighborhood Safety/Crime Survey Modified from PhenX (NSC): 1. I feel safe walking in the neighborhood; 2. Violence is not a problem in my neighborhood; 3.My neighborhood is safe from crime.<br><br>The questionnaire measured neighborhood risk and protective factors, crime. The neighborhood area was defined as “a 20-minute walk (or about a mile) from home”, and parents were asked to score the questions from 1 to 5 indicating that indicate that they: 1 = Strongly Disagree; 2 = Disagree; 3 = Neutral (neither agree nor disagree); 4 = Agree; 5 = Strongly Agree.	Derived from 3 elements of the survey of the ABCD Parent Neighborhood Safety/Crime Survey Modified from PhenX (NSC).
(3) Other		
reshist_addr1_popdensity	United Nations (UN) adjusted population density.	Residential history derived and based on population counts of the 2010 census tract while adjusted based on potential underreporting across the world (United Nation adjusted). Grid version was sourced from the Socioeconomic Data and Applications Center (SEDAC) [31].
reshist_addr1_proxrd	Proximity to major roads, in meters. Calculated bins of road proximity (meters) includes: 1= <500 meters; 2= 5-1000 meters; 3= 1000-1500 meters; 4=1500-5000 meters; 5= >5000 meters	Residential history derived, proximity to major roads and highways, in meters, sourced from the U.S. Geological Survey [32].
mri_info_manufacturer	Imaging device manufacturer.	ABCD MRI Scanner Information.
dmri_dti_mean.motion<br>dmri_rsi_mean.motion	ABCD dMRI DTI Part 1: Average framewise displacement in mm.	Average framewise displacement in mm.
ehi_ss_score	Youth Edinburgh Handedness Inventory Short Form (EHIS) – Handedness score rating	Handedness, laterality quotient – takes the mean during activities of Writing, Throwing Toothbrush Spoon, coded into the following dummy codes:<br><br>1 = right handed; 2 = left handed; 3=mixed
hemisphere	Brain hemisphere: left, right.	-

© 2021 Burnor E et al. *JAMA Network Open.*

eTable 2. Comparison of population characteristics across datasets

	Full ABCD Baseline Dataset (N=11,884)	Analytic Dataset (N=7,602)	p-value[a]	No $PM_{2.5}$ Estimate (n = 698)	Incidental MRI findings (n = 369)	Low Quality/ Missing MRI (n = 2,043)	Removed siblings (n = 1125)
**Sex**			0.851				
Female	5682 (47.8%)	3647 (48.0%)		356 (51.0%)	189 (51.2%)	905 (44.3%)	562 (50.0%)
Male	6196 (52.2%)	3955 (52.0%)		342 (49.0%)	180 (48.8%)	1138 (55.7%)	563 (50.0%)
**Age**			0.301				
Mean (SD)	118.979 (7.496)	119.092 (7.412)		118.536 (7.490)	119.434 (7.428)	118.085 (7.369)	119.812 (8.142)
Range	107.000 - 133.000	107.000 - 133.000		107.000 - 132.000	107.000 - 132.000	107.000 - 132.000	107.000 - 132.000
**Race/ Ethnicity**			**0.03**				
Missing (N)	2	0		0	0	1	1
White	6182 (52.1%)	4025 (52.9%)		302 (43.3%)	218 (59.1%)	915 (44.8%)	703 (62.5%)
Black	1784 (15.0%)	1025 (13.5%)		165 (23.6%)	49 (13.3%)	418 (20.5%)	119 (10.6%)
Hispanic	2411 (20.3%)	1618 (21.3%)		144 (20.6%)	66 (17.9%)	407 (19.9%)	168 (14.9%)
Asian	252 (2.1%)	160 (2.1%)		11 (1.6%)	10 (2.7%)	58 (2.8%)	11 (1.0%)
Other	1247 (10.5%)	774 (10.2%)		76 (10.9%)	26 (7.0%)	244 (11.9%)	123 (10.9%)
**Family Income**			0.293				
Missing	2	0		0	1	1	0
< 50k	3224 (27.1%)	1976 (26.0%)		209 (29.9%)	91 (24.7%)	690 (33.8%)	248 (22.0%)
>=50k & < 100k	3071 (25.9%)	1987 (26.1%)		165 (23.6%)	85 (23.1%)	528 (25.9%)	298 (26.5%)
>= 100k	4565 (38.4%)	2998 (39.4%)		242 (34.7%)	159 (43.2%)	635 (31.1%)	510 (45.3%)
Don't know or refuse	1016 (8.6%)	641 (8.4%)		82 (11.7%)	33 (9.0%)	189 (9.3%)	69 (6.1%)

© 2021 Burnor E et al. *JAMA Network Open.*

	Full ABCD Baseline Dataset (N= 11,884)	Analytic Dataset (N= 7,602)	p-value [a]	No PM2.5 Estimate (n = 698)	Incidental MRI findings (n = 369)	Low Quality/ Missing MRI (n = 2,043)	Removed siblings (n = 1125)
**Highest Household Education**			0.253				
Missing (N)	14	8		2	0	3	1
< HS Diploma	593 (5.0%)	358 (4.7%)		52 (7.5%)	18 (4.9%)	130 (6.4%)	33 (2.9%)
HS Diploma/GED	1132 (9.5%)	676 (8.9%)		83 (11.9%)	33 (8.9%)	262 (12.8%)	76 (6.8%)
Some college:	3080 (26.0%)	1937 (25.5%)		188 (27.0%)	76 (20.6%)	600 (29.4%)	271 (24.1%)
Bachelor:	3015 (25.4%)	1938 (25.5%)		157 (22.6%)	99 (26.8%)	468 (22.9%)	343 (30.5%)
Post Graduate:	4044 (34.1%)	2685 (35.4%)		216 (31.0%)	143 (38.8%)	580 (28.4%)	401 (35.7%)
**Parental Employment Status**			0.479				
Missing (N)	56	33		5	1	11	6
Working	8218 (69.5%)	5315 (70.2%)		472 (68.1%)	239 (64.9%)	1365 (67.2%)	798 (71.3%)
Unemployed	674 (5.7%)	407 (5.4%)		59 (8.5%)	12 (3.3%)	156 (7.7%)	35 (3.1%)
Other	2930 (24.8%)	1847 (24.4%)		162 (23.4%)	117 (31.8%)	511 (25.1%)	286 (25.6%)
**Perceived Neighborhood Safety**	**Safety**		0.901				
Missing	8	1		1	1	5	0
Mean (SD)	3.889 (0.976)	3.887 (0.968)		3.861 (1.031)	4.015 (0.913)	3.803 (1.022)	4.043 (0.902)
Range	1.000 - 5.000	1.000 - 5.000		1.000 - 5.000	1.000 - 5.000	1.000 - 5.000	1.000 - 5.000
**Handedness**			0.37				
Right	9429 (79.4%)	6097 (80.2%)		549 (78.7%)	279 (75.6%)	1572 (76.9%)	898 (79.8%)
Left	848 (7.1%)	527 (6.9%)		43 (6.2%)	39 (10.6%)	153 (7.5%)	81 (7.2%)
Ambidextrous	1601 (13.5%)	978 (12.9%)		106 (15.2%)	51 (13.8%)	318 (15.6%)	146 (13.0%)

© 2021 Burnor E et al. *JAMA Network Open.*

	Full ABCD Baseline Dataset (N= 11,884)	Analytic Dataset (N= 7,602)	p-value [a]	No PM2.5 Estimate (n = 698)	Incidental MRI findings (n = 369)	Low Quality/ Missing MRI (n = 2,043)	Removed siblings (n = 1125)
**MRI Manufacturer**			**< 0.001**				
Missing	288	124		26	6	93	32
GE Medical Systems	2974 (25.7%)	1795 (24.0%)		89 (13.2%)	80 (22.0%)	833 (42.7%)	143 (13.1%)
Philips Medical Systems	1516 (13.1%)	844 (11.3%)		228 (33.9%)	41 (11.3%)	288 (14.8%)	115 (10.5%)
Siemens	7100 (61.3%)	4839 (64.7%)		355 (52.8%)	242 (66.7%)	829 (42.5%)	835 (76.4%)
**Motion (Frame Displacement (mm)**			**< 0.001**				
Missing	800	0		52	0	745	0
Mean (SD)	1.388 (0.576)	1.255 (0.256)		1.445 (0.596)	1.268 (0.248)	2.300 (1.113)	1.231 (0.234)
Range	0.550 - 16.139	0.550 - 1.999		0.686 - 8.774	0.769 - 1.976	0.776 - 16.139	0.614 - 1.997
**$PM_{2.5}$**			0.825				
Missing (N)	700	0		698	0	0	0
Mean (SD)	7.664 (1.562)	7.659 (1.564)		NA	7.378 (1.528)	7.922 (1.538)	7.306 (1.519)
Range	1.720 - 15.900	1.720 - 15.900		NA	3.310 - 13.060	2.370 - 15.900	2.110 - 13.690

[a] P-value from the Pearson *χ-squared* test comparing the distributions of categorical variables between the full ABCD baseline dataset and the final analytic dataset *or* P-value from the ANOVA test comparing means of continuous variables between the full ABCD baseline dataset and the final analytic dataset.

Columns represent the full baseline ABCD dataset, the final analytic dataset, and sub-datasets representing subjects that were removed during data cleaning.

© 2021 Burnor E et al. *JAMA Network Open.*

eTable 3. Distributions of population characteristics at baseline in relation to annual average $PM_{2.5}$.

	Quintiles of PM2.5 (μg/m³)					
	(1.72, 6.3)<br>n = 1526	(6.3, 7.31)<br>n = 1529	(7.31, 8.09)<br>n = 1512	(8.09, 8.85)<br>n = 1518	(8.85, 15.9)<br>n = 1517	P-value[b]
**Sex**						0.289
Female	733 (48.0%)	699 (45.7%)	731 (48.3%)	752 (49.5%)	715 (47.1%)	
Male	793 (52.0%)	830 (54.3%)	781 (51.7%)	766 (50.5%)	802 (52.9%)	
**Age (months): mean (SD)**	119.340 (7.501)	119.331 (7.346)	118.944 (7.280)	118.918 (7.391)	118.822 (7.567)	0.153
**Race/Ethnicity[a]**						< 0.001
White	1085 (71.1%)	938 (61.3%)	831 (55.0%)	664 (43.7%)	506 (33.4%)	
Black	103 (6.7%)	184 (12.0%)	213 (14.1%)	238 (15.7%)	287 (18.9%)	
Hispanic	177 (11.6%)	237 (15.5%)	276 (18.3%)	413 (27.2%)	513 (33.8%)	
Asian	28 (1.8%)	25 (1.6%)	42 (2.8%)	26 (1.7%)	40 (2.6%)	
Other	133 (8.7%)	145 (9.5%)	150 (9.9%)	177 (11.7%)	171 (11.3%)	
**Family Income**						< 0.001
[<50k]	248 (16.3%)	283 (18.5%)	395 (26.1%)	464 (30.6%)	587 (38.7%)	
[>=50K & <100K]	394 (25.8%)	423 (27.7%)	409 (27.1%)	399 (26.3%)	363 (23.9%)	
[>=100K]	799 (52.4%)	694 (45.4%)	601 (39.7%)	528 (34.8%)	377 (24.9%)	
[Don't Know or Refuse]	85 (5.6%)	129 (8.4%)	107 (7.1%)	127 (8.4%)	190 (12.5%)	

© 2021 Burnor E et al. *JAMA Network Open.*

	Quintiles of PM2.5 (μg/m³)					
	(1.72, 6.3)<br>n = 1526	(6.3, 7.31)<br>n = 1529	(7.31, 8.09)<br>n = 1512	(8.09, 8.85)<br>n = 1518	(8.85, 15.9)<br>n = 1517	P-value[b]
< HS Diploma	41 (2.7%)	48 (3.1%)	52 (3.4%)	73 (4.8%)	144 (9.5%)	
HS Diploma/GED	85 (5.6%)	110 (7.2%)	110 (7.3%)	148 (9.7%)	220 (14.5%)	
Some College	285 (18.7%)	333 (21.8%)	406 (26.9%)	449 (29.6%)	469 (31.0%)	
Bachelor	432 (28.3%)	431 (28.2%)	400 (26.5%)	351 (23.1%)	322 (21.3%)	
Post Graduate Degree	681 (44.7%)	605 (39.6%)	543 (35.9%)	497 (32.7%)	359 (23.7%)	
**Parental Employment Status**						< 0.001
Working now: FULL TIME/PART TIME	1144 (75.1%)	1104 (72.5%)	1091 (72.4%)	1029 (68.1%)	946 (62.9%)	
Unemployed	50 (3.3%)	58 (3.8%)	68 (4.5%)	103 (6.8%)	127 (8.4%)	
Other	329 (21.6%)	361 (23.7%)	348 (23.1%)	380 (25.1%)	430 (28.6%)	
**Handedness**						0.962
Right	1207 (79.1%)	1240 (81.1%)	1215 (80.4%)	1213 (79.9%)	1214 (80.0%)	
Left	113 (7.4%)	98 (6.4%)	102 (6.7%)	109 (7.2%)	102 (6.7%)	
Mixed	206 (13.5%)	191 (12.5%)	195 (12.9%)	196 (12.9%)	201 (13.2%)	

© 2021 Burnor E et al. *JAMA Network Open.*

	Quintiles of PM2.5 (μg/m³)					
	(1.72, 6.3)<br>n = 1526	(6.3, 7.31)<br>n = 1529	(7.31, 8.09)<br>n = 1512	(8.09, 8.85)<br>n = 1518	(8.85, 15.9)<br>n = 1517	P-value[b]
GE Medical Systems	26 (1.7%)	246 (16.1%)	474 (31.3%)	567 (37.4%)	482 (31.8%)	
Philips Medical Systems	389 (25.5%)	103 (6.7%)	60 (4.0%)	62 (4.1%)	230 (15.2%)	
Siemens	1063 (69.7%)	1135 (74.2%)	967 (64.0%)	876 (57.7%)	790 (52.1%)	
**Neighborhood quality:**<br>**Mean (SD)**	4.105 (0.865)	4.063 (0.896)	3.991 (0.917)	3.792 (0.969)	3.502 (1.042)	
**DMRI**<br>**Mean Motion:**<br>**Mean (SD)**	1.254 (0.227)	1.217 (0.238)	1.218 (0.263)	1.249 (0.273)	1.339 (0.262)	< 0.001

Note: Percentages are indicated row-wise.
[a] The "Other" race/ethnicity category includes subjects who were parent-identified as American Indian/Native American, Alaska Native, Native Hawaiian, Guamanian, Samoan, Other Pacific Islander, Asian Indian, Chinese, Filipino, Japanese, Korean, Vietnamese, Other Asian, or Other Race
[b] P-value from the Pearson *χ-squared* test comparing the quintile distribution of $PM_{2.5}$ across categorical variables.
P-value from the ANOVA test comparing means of continuous variables across the quintiles of $PM_{2.5}$.

© 2021 Burnor E et al. *JAMA Network Open.*

eTable 4. $PM_{2.5}$-by-hemisphere Analyses for RSI outcomes.

	rN0			rND			hD		
**Tracts**	**Marginal R^2**	**Conditional R^2**	**p-value**	**Marginal R^2**	**Conditional R^2**	**p-value**	**Marginal R^2**	**Conditional R^2**	**p-value**
ATR	0.1596	0.8859	< 0.000	0.381	0.8142	< 0.000	0.2283	0.8082	< 0.000
CGC	0.1897	0.8236	0.014	0.4175	0.7176	< 0.000	0.3327	0.7549	< 0.000
CGH	0.1662	0.784	< 0.000	0.3628	0.7786	< 0.000	0.2983	0.8052	< 0.000
CST	0.2692	0.9005	< 0.000	0.2869	0.8392	0.0026	0.1887	0.8727	< 0.000
FX	0.1496	0.8357	< 0.000	0.1736	0.7895	0.1906	0.0939	0.7745	0.0804
IFO	0.1248	0.869	< 0.000	0.4453	0.8832	< 0.000	0.3097	0.8903	< 0.000
ILF	0.083	0.8401	< 0.000	0.2808	0.833	< 0.000	0.1556	0.8565	< 0.000
SLF	0.1323	0.8924	< 0.000	0.3585	0.8791	< 0.000	0.1875	0.8902	< 0.000
UNC	0.0627	0.8221	< 0.000	0.417	0.834	< 0.000	0.3131	0.8349	< 0.000
All Fibers	0.1127	0.9563	< 0.000	0.4695	0.9686	< 0.000	0.2683	0.9696	< 0.000

R^2 values and FDR-corrected P-values resulting from Type III Analysis of variance (Satterthwaite Method) for RSI measures

© 2021 Burnor E et al. *JAMA Network Open.*

eTable 5. Percent change in RSI rN0 across values of $PM_{2.5}$ and sociodemographic characteristics for significant hemisphere-specific models

Outcome	Tract	Hemisphere	Predictor	Levels	Percent Change	95% CI
RSI rN0	ALLFIB	Left	$PM_{2.5}$	4 μg/m^3 to 8 μg/m^3	0.391	[-0.896, 1.678]
				8 μg/m^3 to 12 μg/m^3	0.958	[-0.344, 2.261]
			Age	6-month increase	0.767	[-0.357, 1.892]
			Income	Less than $50k vs. $50K - $100K	-0.027	[-1.169, 1.115]
				$50K - $100K vs. Greater than $100K	-0.326	[-1.473, 0.821]
	CGH	Left	$PM_{2.5}$	4 μg/m^3 to 8 μg/m^3	1.443	[-0.217, 3.102]
				8 μg/m^3 to 12 μg/m^3	2.164	[0.49, 3.838]
			Age	6-month increase	1.259	[0.108, 2.409]
			Income	Less than $50k vs. $50K - $100K	0.096	[-1.132, 1.325]
				$50K - $100K vs. Greater than $100K	-0.459	[-1.707, 0.789]
	FX	Left	$PM_{2.5}$	4 μg/m^3 to 8 μg/m^3	0.25	[-3.08, 3.58]
				8 μg/m^3 to 12 μg/m^3	3.006	[-0.39, 6.402]
			Age	6-month increase	0.566	[-2.444, 3.576]
			Income	Less than $50k vs. $50K - $100K	-0.327	[-3.357, 2.703]
				$50K - $100K vs. Greater than $100K	0.037	[-3.022, 3.096]
	SLF	Left	$PM_{2.5}$	4 μg/m^3 to 8 μg/m^3	0.658	[-0.359, 1.675]
				8 μg/m^3 to 12 μg/m^3	0.933	[-0.1, 1.966]
			Age	6-month increase	0.778	[0.009, 1.546]
			Income	Less than $50k vs. $50K - $100K	-0.156	[-0.958, 0.646]
				$50K - $100K vs. Greater than $100K	-0.316	[-1.13, 0.498]
	UNC	Left	$PM_{2.5}$	4 μg/m^3 to 8 μg/m^3	0.373	[-1.112, 1.857]
				8 μg/m^3 to 12 μg/m^3	1.949	[0.43, 3.468]
			Age	6-month increase	1.006	[-0.133, 2.146]
			Income	Less than $50k vs. $50K - $100K	-0.027	[-1.214, 1.161]
				$50K - $100K vs. Greater than $100K	-0.527	[-1.727, 0.673]
	FX	Right	$PM_{2.5}$	4 μg/m^3 to 8 μg/m^3	0.507	[-2.296, 3.31]
				8 μg/m^3 to 12 μg/m^3	2.128	[-0.716, 4.972]
			Age	6-month increase	0.681	[-1.753, 3.114]
			Income	Less than $50k vs. $50K - $100K	0.073	[-2.404, 2.55]
				$50K - $100K vs. Greater than $100K	-0.093	[-2.581, 2.395]

© 2021 Burnor E et al. *JAMA Network Open.*

Outcome	Tract	Hemisphere	Predictor	Levels	Percent Change	95% CI
	UNC	Right	$PM_{2.5}$	4 µg/m^3 to 8 µg/m^3	0.541	[-1.094, 2.177]
				8 µg/m^3 to 12 µg/m^3	1.676	[0.014, 3.338]
			Age	6-month increase	1.046	[-0.288, 2.379]
			Income	Less than $50k vs. $50K - $100K	0.198	[-1.174, 1.569]
				$50K - $100K vs. Greater than $100K	-0.764	[-2.139, 0.611]

© 2021 Burnor E et al. *JAMA Network Open*.

eTable 6. $PM_{2.5}$-by-hemisphere Analyses for DTI outcomes.

	FA			MD		
	**Marginal R^2**	**Conditional R^2**	**p-value**	**Marginal R^2**	**Conditional R^2**	**p-value**
ATR	0.4944	0.8724	< 0.000	0.5400	0.9147	< 0.000
CGC	0.5050	0.774	< 0.000	0.4559	0.8394	< 0.000
CGH	0.5336	0.8282	< 0.000	0.6355	0.9221	< 0.000
CST	0.4082	0.8677	0.003	0.6673	0.9662	< 0.000
FX	0.5033	0.8665	< 0.000	0.6280	0.948	< 0.000
IFO	0.5663	0.9111	< 0.000	0.4814	0.9406	< 0.000
ILF	0.4515	0.8377	0.02	0.2934	0.9057	< 0.000
SLF	0.4900	0.8751	< 0.000	0.1998	0.9132	< 0.000
UNC	0.5181	0.8638	< 0.000	0.4008	0.8951	< 0.000
All Fibers	0.6404	0.9788	< 0.000	0.5133	0.9841	< 0.000

R^2 values and FDR-corrected P-values resulting from Type III Analysis of variance (Satterthwaite Method) for DTI measure

© 2021 Burnor E et al. *JAMA Network Open.*

eTable 7. Percent change in DTI MD across values of $PM_{2.5}$ and sociodemographic characteristics for significant hemisphere-specific models

Outcome	Tract	Hemisphere	Predictor	Levels	Percent Change	95% CI
DTI MD	Mean Across All Fibers	Left	$PM_{2.5}$	4 μg/m^3 to 8 μg/m^3	-0.223	[-1.198, 0.751]
				8 μg/m^3 to 12 μg/m^3	-0.814	[-1.8, 0.173]
			Age	6-month increase	-0.372	[-1.255, 0.51]
			Household Income	Less than $50k vs. $50K - $100K	-0.035	[-0.934, 0.864]
				$50K - $100K vs. Greater than $100K	0.209	[-0.695, 1.113]
	Mean Across All Fibers	Right	$PM_{2.5}$	4 μg/m^3 to 8 μg/m^3	-0.201	[-0.935, 0.533]
				8 μg/m^3 to 12 μg/m^3	-0.564	[-1.309, 0.181]
			Age	6-month increase	-0.432	[-1.08, 0.216]
			Household Income	Less than $50k vs. $50K - $100K	-0.065	[-0.727, 0.597]
				$50K - $100K vs. Greater than $100K	0.211	[-0.457, 0.878]
	ATR	Left	$PM_{2.5}$	4 μg/m^3 to 8 μg/m^3	-0.055	[-0.944, 0.833]
				8 μg/m^3 to 12 μg/m^3	-0.774	[-1.67, 0.121]
			Age	6-month increase	-0.41	[-1.22, 0.401]
			Household Income	Less than $50k vs. $50K - $100K	-0.095	[-0.919, 0.728]
				$50K - $100K vs. Greater than $100K	0.204	[-0.624, 1.033]
	CGH	Left	$PM_{2.5}$	4 μg/m^3 to 8 μg/m^3	-0.245	[-1.018, 0.528]
				8 μg/m^3 to 12 μg/m^3	-1.056	[-1.845, -0.268]
			Age	6-month increase	-0.425	[-1.062, 0.212]
			Household Income	Less than $50k vs. $50K - $100K	-0.08	[-0.74, 0.58]
				$50K - $100K vs. Greater than $100K	0.139	[-0.528, 0.806]
	CGH	Right	$PM_{2.5}$	4 μg/m^3 to 8 μg/m^3	-0.013	[-0.766, 0.740]
				8 μg/m^3 to 12 μg/m^3	-0.714	[-1.48, 0.052]
			Age	6-month increase	-0.415	[-1.039, 0.209]
			Household Income	Less than $50k vs. $50K - $100K	-0.037	[-0.683, 0.608]
				$50K - $100K vs. Greater than $100K	0.087	[-0.565, 0.738]

Outcome	Tract	Hemisphere	Predictor	Levels	Percent Change	95% CI
	FX	Left	$PM_{2.5}$	4 µg/m^3 to 8 µg/m^3	-0.141	[-1.298, 1.016]
				8 µg/m^3 to 12 µg/m^3	-1.03	[-2.194, 0.135]
			Age	6-month increase	-0.234	[-1.312, 0.843]
			Household Income	Less than $50k vs. $50K - $100K	0.065	[-1.027, 1.157]
				$50K - $100K vs. Greater than $100K	-0.016	[-1.111, 1.078]
	FX	Right	$PM_{2.5}$	4 µg/m^3 to 8 µg/m^3	-0.184	[-1.31, 0.942]
				8 µg/m^3 to 12 µg/m^3	-0.782	[-1.919, 0.354]
			Age	6-month increase	-0.254	[-1.293, 0.785]
			Household Income	Less than $50k vs. $50K - $100K	-0.012	[-1.066, 1.041]
				$50K - $100K vs. Greater than $100K	0.008	[-1.049, 1.066]
	IFO	Left	$PM_{2.5}$	4 µg/m^3 to 8 µg/m^3	-0.263	[-1.082, 0.557]
				8 µg/m^3 to 12 µg/m^3	-0.637	[-1.469, 0.195]
			Age	6-month increase	-0.396	[-1.123, 0.332]
			Household Income	Less than $50k vs. $50K - $100K	-0.013	[-0.756, 0.731]
				$50K - $100K vs. Greater than $100K	0.213	[-0.536, 0.961]
	ILF	Left	$PM_{2.5}$	4 µg/m^3 to 8 µg/m^3	-0.347	[-1.036, 0.342]
				8 µg/m^3 to 12 µg/m^3	-0.73	[-1.44, -0.021]
			Age	6-month increase	-0.45	[-0.97, 0.070]
			Household Income	Less than $50k vs. $50K - $100K	0.003	[-0.548, 0.553]
				$50K - $100K vs. Greater than $100K	0.192	[-0.367, 0.751]
	ILF	Right	$PM_{2.5}$	4 µg/m^3 to 8 µg/m^3	-0.43	[-1.128, 0.268]
				8 µg/m^3 to 12 µg/m^3	-0.593	[-1.312, 0.126]
			Age	6-month increase	-0.494	[-1.032, 0.044]
			Household Income	Less than $50k vs. $50K - $100K	-0.114	[-0.68, 0.452]
				$50K - $100K vs. Greater than $100K	0.293	[-0.282, 0.868]
	SLF	Left	$PM_{2.5}$	4 µg/m^3 to 8 µg/m^3	-0.422	[-1.077, 0.232]
				8 µg/m^3 to 12 µg/m^3	-0.714	[-1.388, -0.039]
			Age	6-month increase	-0.526	[-1.024, -0.029]
			Household Income	Less than $50k vs. $50K - $100K	0.01	[-0.516, 0.536]
				$50K - $100K vs. Greater than $100K	0.198	[-0.336, 0.732]

© 2021 Burnor E et al. *JAMA Network Open.*

Outcome	Tract	Hemisphere	Predictor	Levels	Percent Change	95% CI
	UNC	Left	$PM_{2.5}$	4 μg/m^3 to 8 μg/m^3	-0.171	[-0.909, 0.566]
				8 μg/m^3 to 12 μg/m^3	-0.981	[-1.732, -0.23]
			Age	6-month increase	-0.439	[-1.047, 0.168]
			Household Income	Less than $50k vs. $50K - $100K	-0.002	[-0.632, 0.628]
				$50K - $100K vs. Greater than $100K	0.192	[-0.444, 0.828]
	UNC	Right	$PM_{2.5}$	4 μg/m^3 to 8 μg/m^3	-0.178	[-1.019, 0.663]
				8 μg/m^3 to 12 μg/m^3	-0.828	[-1.682, 0.025]
			Age	6-month increase	-0.446	[-1.181, 0.29]
			Household Income	Less than $50k vs. $50K - $100K	-0.126	[-0.879, 0.627]
				$50K - $100K vs. Greater than $100K	0.303	[-0.457, 1.063]
	CC		$PM_{2.5}$	4 μg/m^3 to 8 μg/m^3	-0.223	[-1.198, 0.751]
				8 μg/m^3 to 12 μg/m^3	-0.814	[-1.8, 0.173]
			Age	6-month increase	-0.372	[-1.255, 0.51]
			Household Income	Less than $50k vs. $50K - $100K	-0.035	[-0.934, 0.864]
				$50K - $100K vs. Greater than $100K	0.209	[-0.695, 1.113]

© 2021 Burnor E et al. *JAMA Network Open.*

References

1. Bagot KS, Matthews SA, Mason M, et al. Current, future and potential use of mobile and wearable technologies and social media data in the ABCD study to increase understanding of contributors to child health. *Dev Cogn Neurosci.* 2018;32:121-129.
2. Casey BJ, Cannonier T, Conley MI, et al. The Adolescent Brain Cognitive Development (ABCD) study: Imaging acquisition across 21 sites. *Dev Cogn Neurosci.* 2018;32:43-54.
3. Feldstein Ewing SW, Chang L, Cottler LB, Tapert SF, Dowling GJ, Brown SA. Approaching Retention within the ABCD Study. *Dev Cogn Neurosci.* 2018;32:130-137.
4. Garavan H, Bartsch H, Conway K, et al. Recruiting the ABCD sample: Design considerations and procedures. *Dev Cogn Neurosci.* 2018;32:16-22.
5. Hagler DJ, Hatton SN, Makowski C, et al. Image processing and analysis methods for the Adolescent Brain Cognitive Development Study. *bioRxiv.* 2018.
6. Luciana M, Bjork JM, Nagel BJ, et al. Adolescent neurocognitive development and impacts of substance use: Overview of the adolescent brain cognitive development (ABCD) baseline neurocognition battery. *Dev Cogn Neurosci.* 2018;32:67-79.
7. Uban KA, Horton MK, Jacobus J, et al. Biospecimens and the ABCD study: Rationale, methods of collection, measurement and early data. *Dev Cogn Neurosci.* 2018;32:97-106.
8. Michelini G, Barch DM, Tian Y, Watson D, Klein DN, Kotov R. Delineating and validating higher-order dimensions of psychopathology in the Adolescent Brain Cognitive Development (ABCD) study. *Translational Psychiatry.* 2019;9(261).
9. Cserbik D, Chen JC, McConnell R, et al. Fine particulate matter exposure during childhood relates to hemispheric-specific differences in brain structure. *Environment International.* 2020;143:105933-105933.
10. Li Y, Thompson W, Reuter C, et al. Rates of Incidental Findings in Brain Magnetic Resonance Imaging in Children. *JAMA Neurology.* 2021;78(5):578-587.
11. Hagler DJ, Jr., Ahmadi ME, Kuperman J, et al. Automated white-matter tractography using a probabilistic diffusion tensor atlas: Application to temporal lobe epilepsy. *Hum Brain Mapp.* 2009;30(5):1535-1547.
12. Hagler DJ, Jr., Hatton S, Cornejo MD, et al. Image processing and analysis methods for the Adolescent Brain Cognitive Development Study. *Neuroimage.* 2019;202:116091.
13. Zhuang J, Hrabe J, Kangarlu A, et al. Correction of eddy-current distortions in diffusion tensor images using the known directions and strengths of diffusion gradients. *J Magn Reson Imaging.* 2006;24(5):1188-1193.
14. Leemans A, Jones DK. The B-matrix must be rotated when correcting for subject motion in DTI data. *Magn Reson Med.* 2009;61(6):1336-1349.
15. Chang LC, Jones DK, Pierpaoli C. RESTORE: robust estimation of tensors by outlier rejection. *Magn Reson Med.* 2005;53(5):1088-1095.
16. Holland D, Kuperman JM, Dale AM. Efficient correction of inhomogeneous static magnetic field-induced distortion in Echo Planar Imaging. *Neuroimage.* 2010;50(1):175-183.
17. Jovicich J, Czanner S, Greve D, et al. Reliability in multi-site structural MRI studies: effects of gradient non-linearity correction on phantom and human data. *Neuroimage.* 2006;30(2):436-443.
18. Wells WM, 3rd, Viola P, Atsumi H, Nakajima S, Kikinis R. Multi-modal volume registration by maximization of mutual information. *Med Image Anal.* 1996;1(1):35-51.

© 2021 Burnor E et al. *JAMA Network Open.*

19. Friston K. J. AJ, Frith C. D., Poline J. P., Heather J. D., Frackowiak R. S. . Spatial registration and normalization of images. *Hum Brain Mapp.* 1995;2:165-189.
20. Fischl B SD, Busa E, Albert M, Dieterich M, Haselgrove C, van der Kouwe A, Killiany R, Kennedy D, Klaveness S, Montillo A, Makris N, Rosen B, Dale AM. Whole brain segmentation: automated labeling of neuroanatomical structures in the human brain. *Neuron.* 2002;33:341-355.
21. White NS, Leergaard TB, D'Arceuil H, Bjaalie JG, Dale AM. Probing tissue microstructure with restriction spectrum imaging: Histological and theoretical validation. *Hum Brain Mapp.* 2013;34(2):327-346.
22. White NS, McDonald C, Farid N, et al. Diffusion-weighted imaging in cancer: physical foundations and applications of restriction spectrum imaging. *Cancer Res.* 2014;74(17):4638-4652.
23. White NS, McDonald CR, Farid N, Kuperman JM, Kesari S, Dale AM. Improved conspicuity and delineation of high-grade primary and metastatic brain tumors using "restriction spectrum imaging": quantitative comparison with high B-value DWI and ADC. *AJNR Am J Neuroradiol.* 2013;34(5):958-964, S951.
24. Basser PJ, Mattiello J, LeBihan D. MR diffusion tensor spectroscopy and imaging. *Biophys J.* 1994;66(1):259-267.
25. Greenland S, Brumback B. An overview of relations among causal modelling methods. *Int J Epidemiol.* 2002;31(5):1030-1037.
26. Weng HY, Hsueh YH, Messam LL, Hertz-Picciotto I. Methods of covariate selection: directed acyclic graphs and the change-in-estimate procedure. *Am J Epidemiol.* 2009;169(10):1182-1190.
27. Bell ML, Ebisu K. Environmental inequality in exposures to airborne particulate matter components in the United States. *Environmental Health Perspectives.* 2012;120(12):1699-1704.
28. Miranda ML, Edwards SE, Keating MH, Paul CJ. Making the Environmental Justice Grade: The Relative Burden of Air Pollution Exposure in the United States. *Int J Environ Res Public Health.* 2011;8(6):1755-1771.
29. Hajat A, Hsia C, O'Neill MS. Socioeconomic Disparities and Air Pollution Exposure: a Global Review. *Curr Environ Health Rep.* 2015;2(4):440-450.
30. Mujahid MS, Diez Roux AV, Morenoff JD, Raghunathan T. Assessing the measurement properties of neighborhood scales: from psychometrics to ecometrics. *Am J Epidemiol.* 2007;165(8):858-867.
31. Center for International Earth Science Information Network - CIESIN - Columbia University. Gridded Population of the World, Version 4 (GPWv4): Population Density Adjusted to Match 2015 Revision UN WPP Country Totals. In. Palisades, NY: NASA Socioeconomic Data and Applications Center (SEDAC); 2016.
32. U.S. Department of the Interior. One Million-Scale Major Roads of the United States. http://nationalmap.gov/small_scale/mld/1roadsl.html. Published 2017. Accessed.
33. Karner AA, Eisinger DS, Niemeier DA. Near-roadway air quality: synthesizing the findings from real-world data. *Environ Sci Technol.* 2010;44(14):5334-5344.
34. Bureau USC. Percent Changes https://www2.census.gov/programs-surveys/acs/tech_docs/accuracy/percchg.pdf. Published 2015. Accessed November 5, 2020, 2020.

© 2021 Burnor E et al. *JAMA Network Open.*
